# Going beyond the DSM in predicting, diagnosing, and treating autism spectrum disorder with covarying alexithymia and OCD: A structural equation model and process-based predictive coding account

**DOI:** 10.3389/fpsyg.2022.993381

**Published:** 2022-09-06

**Authors:** Darren J. Edwards

**Affiliations:** Department of Public Health, Swansea University, Swansea, United Kingdom

**Keywords:** autism (ASD), alexithymia, obsessive-compulsive disorder (OCD), structural equation model (SEM), graph theory, psychological flexibility

## Abstract

**Background:**

There is much overlap among the symptomology of autistic spectrum disorders (ASDs), obsessive compulsive disorders (OCDs), and alexithymia, which all typically involve impaired social interactions, repetitive impulsive behaviors, problems with communication, and mental health.

**Aim:**

This study aimed to identify direct and indirect associations among alexithymia, OCD, cardiac interoception, psychological inflexibility, and self-as-context, with the DV ASD and depression, while controlling for vagal related aging.

**Methodology:**

The data involved electrocardiogram (ECG) heart rate variability (HRV) and questionnaire data. In total, 1,089 participant's data of ECG recordings of healthy resting state HRV were recorded and grouped into age categories. In addition to this, another 224 participants completed an online survey that included the following questionnaires: Yale-Brown Obsessive Compulsive Scale (Y-BOCS); Toronto Alexithymia Scale 20 (TAS-20); Acceptance and Action Questionnaire (AAQII); Depression, Anxiety, and Stress Scale 21 (DAS21); Multi-dimensional Assessment of Interoceptive Awareness Scale (MAIA); and the Self-as-Context Scale (SAC).

**Results:**

Heart rate variability was shown to decrease with age when controlling for BMI and gender. In the two SEMs produced, it was found that OCD and alexithymia were causally associated with autism and depression indirectly through psychological inflexibility, SAC, and ISen interoception.

**Conclusion:**

The results are discussed in relation to the limitations of the DSM with its categorical focus of protocols for syndromes and provide support for more flexible ideographic approaches in diagnosing and treating mental health and autism within the Extended Evolutionary Meta-Model (EEMM). Graph theory approaches are discussed in their capacity to depict the processes of change potentially even at the level of the relational frame.

## Introduction

The Diagnostic and Statistical Manuel 5th Edition (DSM-5) (American Psychiatric Association, 2013a) describes autistic spectrum disorders (ASDs) as involving impaired social interactions, repetitive behaviors, and problems with communication. Additional features can include poor eye contact (Esposito and Venuti, 2008), sensory modulatory dysfunction (Baranek et al., 2005), motor dysfunction (Ozonoff et al., 2008), and cognitive developmental problems (Esposito et al., 2009; Edwards et al., 2012).

There have been several potential advantages of the DSM classification suggested for disorders, such as ASD, as it attempts to standardize diagnosis and treatment across different treatment providers, provides a quick guide, and should eliminate guesswork (Möller, 2014). This should, in principle, provide a useful guide for both clinicians and researchers; however, the DSM classification approach also presents many disadvantages. One such disadvantage is that there may be issues with such a rigid classification taxonomy as the DSM adopts a latent disease model that is suggested by the American Psychiatric Association (2013a,b) and assumes a medical illness model. This approach assumes that symptoms should reflect some specific sets of underlying and latent disease entities; however, latent categories are often not distinct. This can be found in the evidence which has found a high degree of comorbidity between these latent categories, indicating that these categorical clusters of symptoms advocated by the DSM are not linked to discrete functions as they were intended to do (Kupfer et al., 2008). Despite many attempts to overcome the problem of comorbidity, the DSM still does not provide clinicians and researchers with any treatment-specific indications for those cases presenting with psychiatric comorbidity (Dell'Osso and Pini, 2012). These type of problems have led to growing disinterest in the most recent version, the DSM-5 (Hayes et al., 2019).

Such growing disagreement with this rigid classification protocol approach for some times has led to calls for a move toward a process-based approach in the diagnostics and treatment of mental health disorders (Forgeard et al., 2011). This has been most recently formalized in a process-based therapy (PBT) approach, which advocates that diagnostic and therapeutic efforts should focus more on the causal processes that underlie the efforts to produce change and not through rigid categories advocated by the DSM (Hayes and Hofmann, 2017, 2018; Hofmann and Hayes, 2019; Hofmann et al., 2021). PBT research emphasizes the Extended Evolutionary Meta Model (EEMM), which highlights the processes of change as multi-level and multi-dimensional rather than singularly focused through protocols of syndrome as suggested by the DSM. EEMM specifies six dimensions at the individual level where processes of change can occur; affective (positive and negative affects), cognitive (problem-solving skills, reappraisal, etc.), attentional (mindfulness, noticing in the present moment, awareness of feelings), self (self-esteem, self-efficacy, self-compassion, and self-as-context), motivation (individual goals and values), and overt behavior (striving and commitment to goals). There are two additional levels in this model, that being the physiological level (such as autonomic functioning, predictive coding, interoception of the insular cortex), and social level (such as group motivations and values).

This problem of comorbidity when attempting to strictly classify disorders can be seen clearly in many clinical cases. For example, there are several autistic symptoms that have similarities and overlap with the symptoms in other clinically diagnosable conditions, such as schizophrenia, such that both conditions involve problems with emotional functioning and information processing (Lewis and Levitt, 2002; Volkmar et al., 2004). Indeed, evidence suggests that ASD and schizophrenia may share some commonalities in phenomenology (Couture et al., 2010), in biological pathway pathophysiology (Pinkham et al., 2008; Guilmatre et al., 2009; Sugranyes et al., 2011), and in treatment (McCracken et al., 2002). However, these two conditions have been clearly delineated for their unique aspects for many years (Kanner, 1965).

Such delineation has been more difficult when comparing conditions such as ASD and alexithymia (Kinnaird et al., 2019). Alexithymia, which is regarded as a personality trait and relates to the difficulties in emotional processing, such as recognizing emotions, has also been reported in ASD (Silani et al., 2008; Guastella et al., 2010). More specifically, it has been suggested that problems with emotional processing observed in ASD stem from co-occurring alexithymia (Bird and Cook, 2013; Cook and Shah, 2013; Brewer et al., 2015). Alexithymia was first recognized in the 1970s and has been described as a condition involving difficulties in recognizing and distinguishing different emotions, a lack of imagination, and thoughts centered on the external world rather than internal world (Sifneos, 1973). Though not always identified in individuals who have a diagnosis of ASD, alexithymia has been found to be more extreme in ASD individuals compared to the general population (Hill et al., 2004; Berthoz and Hill, 2005; Fitzgerald and Bellgrove, 2006). Indeed, there is a growing body of research, which suggests that the problems associated with emotional processing in ASD individuals are driven by alexithymia. Evidence supporting this comes from studies that have shown, for instance, that when controlling for alexithymia and autism, it is the levels of alexithymia and not autism which predict problems with facial, vocal, and music emotional recognition (Heaton et al., 2012; Allen et al., 2013; Cook and Shah, 2013). A meta-analysis (Kinnaird et al., 2019) identified 15 studies where alexithymia was found to be higher in an ASD population when compared to a neurotypical population (49.93 compared to 4.89%). They concluded that alexithymia represents a specific subgroup in ASD, and that further research was needed to understand the nature and implications of co-occurring comorbid alexithymia in ASD. This comorbidity is clearly a problem for the DSM, which favors distinct categories.

Another connection that is difficult to delineate is the connection between ASD (including with alexithymia) and obsessive compulsive disorder (OCD) symptoms. For example, ASD traits (such as ritualistic behavior, attachment to objects, and in some cases low levels of sociability) have been identified in individuals with OCD (Ivarsson and Melin, 2008). There are also substantial similarities and overlap in the pathophysiology between OCD and ASD (Meier et al., 2015), though less is known about the clinical etiological cohesion between these disorders. Furthermore, it has been found that individuals with OCD had significantly higher levels of alexithymia than those who did not (Grabe et al., 2006), which echoes the comorbidity problem that ASD has.

Less work has linked ASD with alexithymia and OCD concretely. Some of the work in ASD have speculated; for instance, that deficits in localized areas of basal ganglia and cerebellum abnormalities (Nayate et al., 2005; Rinehart et al., 2006; Qiu et al., 2010) are responsible for gait function, movement problems, and also social and communication issues, which may also be applicable to explain alexithymia and OCD symptoms in some cases, but not all. However, one particularly interesting and promising psychophysiological pathway that may prove to be a crucial link to explain the comorbidity among alexithymia, ASD, and OCD is the interoceptive pathway.

Interoception is the representation of the internal system at any given moment. It is formed in the brain (primarily the insular cortex) by interpreting signals received from the bodily organs *via* general visceral afferent neurons (found in cranial nerves VII, IX, and X) of the autonomic nervous system (ANS) (Cameron, 2001; Craig, 2002). The interoceptive pathway consists of unmyelinated C and myelinated Aδ afferent fibers which transmit a signal from the laminae I vertebra of the spine to the posterior gray column of the spinal column and then to the hypothalamus, anterior insular, and cingulate cortices (Pollatos et al., 2007; Craig, 2008). These brain areas receive information signals from viscera, thermoregulatory, nociceptive, and endocrine systems (Pollatos et al., 2007). Once the brain has received these signals, it then integrates this information in terms of their varying motivational immediacy, such as determining whether to respond to sensations, such as prickly burning pain, warmth-coldness, hunger, need to urinate, and sensual touch (Strigo and Craig, 2016). The signals are then further processed for motivational and emotional relevant behaviors, through organizing it within primary emotional and motivational brain regions of the limbic system, the anterior insular, and cingulate cortices of the homeostatic sensorimotor cortex (Murphy et al., 2003; Craig, 2014). It has been suggested that at this stage, a meta-representation of “self” emerges allows for very specific regulatory responses to the signals to emerge.

Interoception is typically measured through an index of interoceptive accuracy (IAc) or interoceptive sensitivity (IS) (these measures are the same), which are recorded through the heartbeat perception task (Schandry, 1981; Pollatos et al., 2009; Mallorquí-Bagué et al., 2014), which asks participants to count their perceived heartbeats, and then, the following formula is applied:


(1) $$\begin{array}{l} 1-\frac{|n\;beats_{actual}-n\;beats_{perceived}|}{\left(n\;beats_{actual}+\;n\;beats_{perceived}\right)/2} \end{array}$$

Another approach is to use the multi-dimensional interoceptive awareness questionnaire (MAIA-2) (Mehling et al., 2018), which assesses a more conscious self-report measure (than the heartbeat perception task) of interoception. There is a distinction between the ability to detect interoceptive information (such as heartbeat detection) in the form of IAc and an individual's conscious beliefs about their interoceptive abilities, called interoceptive sensibility (ISen). The MAIA-2 assessment is a self-report questionnaire measure of ISen (Mehling et al., 2018; Smith et al., 2021), whereas another measure of ISen is a simple confidence measure of how accurate the participants felt they were at the heart beat counting task (Mehling, 2016).

Altered interoception is associated with an increase in psychopathological disorders, such as an increase in chronic pain, anxiety, depression, and eating disorders (Paulus and Stein, 2010; Klabunde et al., 2013; Di Lernia et al., 2016; Duschek et al., 2017), but is not predicative of a specific emotional regulation strategy (Pinna and Edwards, 2020). Typically, lower IAc/IS scores lead to greater pathology, and it is suggested that inefficient or an impaired relay of interoceptive information, transmitting a greater number of interoceptive errors than usual from peripheral structures to higher cortical centers, leads to dysfunctional “body mapping”(Craig, 2008; Harshaw, 2015). This eventually leads to dysfunctional self-regulatory processes and ultimately to aberrant emotional, behavioral, and autonomic sequelae (Craig, 2008).

Increased IAw has been shown to have both positive and negative outcomes. For example, increased focus on physical sensations IAw has been associated with anxiety, hypochondriasis, somatization, and hypervigilance (Paulus and Stein, 2006). It has been suggested that what determines whether increased IAw/ISen leads to maladaptive or healthy outcomes depends on the interoceptive style (Mehling et al., 2018). Mehling et al. suggest that the maladaptive form of IAw/ISen that is associated with the maladaptive outcomes (e.g., of hypervigilance) is anxiety-driven interoception. They suggest that with the emerging field of mindful bodily awareness, maybe a more adaptive form of IAw/ISen can be promoted, rather than that driven by anxiety, which are more likely to lead to more positive mental health outcomes. Some evidence confirm this suggestion, as mindful IAw in the form of a body scan technique (Ussher et al., 2014) has been shown to reduce pain-related distress and the degree to which pain interferes with social relations. Mindful IAw has also shown to reduce stress states in post-traumatic stress disorder (PTSD) and associated depressive symptoms (Colgan et al., 2016).

In autism specifically, interoception appears to be atypical, but the degree and directionality is unclear due to the heterogeneous nature of the condition (DuBois et al., 2016). There have been some controversies as to whether abnormalities in the interoceptive system actually cause autism, with some researchers saying that it does (Quattrocki and Friston, 2014) and others suggesting that it does not (Nicholson et al., 2018). It is perhaps important to note that interoception is not just central to the processing of bodily signals for the requirement of homeostasis and allostasis but also the perception of these bodily signals (Cameron, 2001; Craig, 2002; Critchley et al., 2004). As such, interoception is also central in a range of cognitive and emotional processing which includes memory, decision-making, emotional processing, social interactions, body ownership, a sense of self, and even consciousness (Critchley et al., 2001; Dunn et al., 2010; Shah et al., 2017; Critchley and Garfinkel, 2018; Tsakiris and De Preester, 2018). With such a central system, it is likely that interoception plays a key role in autism and other related conditions.

One of these related conditions is alexithymia, and it has indeed been suggested that alexithymia is the result of impaired interceptive pathway (Herbert et al., 2011; Brewer et al., 2016). For instance, some suggestions are that the interoceptive pathway is thought to relate to developmental dysfunction of the connectivity among the limbic structures, the anterior insular (AntIn), and the anterior cingulate context (ACC) (Lane et al., 1997; Craig, 2002; Singer et al., 2009). This could also mean that alexithymia is the result of dysfunction with the homeostasis mapping of regulatory responses and the generation of a meta representation of “self” that is thought to be an important as part of the interoceptive pathway (Damasio and Carvalho, 2013).

Interoception in the form of IAc has been found to be attenuated in individuals with OCD (Schultchen et al., 2019; Demartini et al., 2021) and did not change over time with standardized cognitive behavioral therapy (CBT). There have been mixed results in relation to whether individuals with OCD have increased or lowered IAc, whereby one study has reported that IAc was higher in OCD than healthy controls (Yoris et al., 2017) and another has indicated that it was lower (Schultchen et al., 2019). In addition to this, an functional magnetic resonance imaging (fMRI) study has shown that sensory phenomena (SP) that are aversive uncomfortable sensations that accompany repetitive behaviors in OCD result in the hyperactivation of the insular (a primary area of interoceptive processing) (Brown et al., 2019). These mixed results may provide further evidence that the type of interoception (e.g., mindfully or anxiety-based) is more important than whether there is a direct increase or decrease in overall interoception. One study specifically focusing on ISen showed that the MAIA subscales were differentially associated with the different OCD symptom dimensions.

Central to interoception is the vagus nerve, which is the main cranial nerve in the human body known to primarily relay visceral (interoceptive) signals to the brain (Critchley and Harrison, 2013; Yoris et al., 2018; Quadt et al., 2019). The vagus nerve is the 10th cranial nerve (labeled CN X) and has a central role in transmitting interoceptive information to the brain, as well as being main anatomical component of the parasympathetic nervous system (PNS) (Walker, 1990). It has been largely acknowledged for its afferent neuron functions that make up 80% of the nerves which relay sensory information from viscero-somatic structures (Porges, 2007; Bonaz et al., 2017). It is assumed that the vagus nerve not only includes visceral homeostatic function, but also is involved in social, cognitive, and affective components (Membrilla et al., 2020). A recent systematic review of the literature showed that both indices of interoception along with more direct vagal activity were shown to be central to emotional regulation (Pinna and Edwards, 2020). For example, the study found that individuals with higher parasympathetic activity and interoception were associated with higher levels of emotional regulation. This, therefore, shows the important role of vagal function and interoception in emotional regulation and mental health.

Vagal tone can be measured in a variety of different ways, all of which involve an electrocardiograph (ECG). A commonly used measure is heart rate variability (HRV), which measures the variability between each consecutive heartbeat, and is described more formally as measuring sequential R-R peaks in the QRS complex of an ECG measure Task Force of the European Society of Cardiology the North American Society of Pacing Electrophysiology (1996). HRV has been found to be a reliable indicator of psychosomatic functions that include emotional and behavioral regulations (Laborde et al., 2018; Pinna and Edwards, 2020). The heart receives dual innervation from the ANS in the form of the sympathetic nervous system (SNS) and PNS. One of the common measures of this HRV construct is the frequency domain index, whereby the high frequency (HF) has been shown to provide a reliable index of vagal function (Thayer, 2009). In addition to the HF index, HRV can be measured using a time domain index called the root mean square successive difference (RMSSDD), as well as the respiratory sinus arrhythmia (RSA) index, which provides a value of HRV in synchrony with the respiratory cycle. A high HRV measure indicates increased parasympathetic activity whereas lower HRV indicates lower parasympathetic and greater sympathetic activity.

One important observation is that HRV has been found to positively correlate with ISen in healthy individuals (Owens et al., 2018). Interestingly, alexithymia has been found to negatively correlate with ISen but not IAc (Zamariola et al., 2018). This link has been supported by another study, which showed that alexithymia was significantly negatively correlated with the dimensions of ISen as recorded by MAIA-2 (noticing, not-distracting, attention regulation, emotional awareness, self-regulation, body listening, and trusting body) (Edwards and Lowe, 2021). Lower HF-HRV has also been found to be associated with higher alexithymia (Lischke et al., 2018), which is consistent with the ISen data, and indicates that these two indices are closely related (both abstracted from the vagal nerve).

In relation to autism, a recent meta-analysis showed that HRV in autism is lower at rest than healthy controls (Cheng et al., 2020); however, ISen (when using the Porges Body Perception Questionnaire) was found to be higher in those with higher autism. For OCD, the results are more mixed. One study has found that OCD showed no difference in HRV when comparing with healthy controls (Slaap et al., 2004); however, another study showed a decrease in HRV for patients with OCD compared to healthy controls (Pittig et al., 2013).

Some researchers have suggested that interoception is a complex and multi-faceted construct which requires continual refinement in conceptualization and operationalizing to fully understand how it can be used to explore psychopathology (Trevisan et al., 2021). Instead of simply concluding that higher or lower interoception is better or worse for mental health, rather a better distinction may be whether the interoceptive bodily awareness is adaptive or maladaptive (Mehling et al., 2018; Trevisan et al., 2021). To conceptually link interoception and vagal functioning (e.g., HRV) in a broader model which is inclusive of ASD, alexithymia, and OCD, existing models can be considered.

Several models exist that explain the biological and brain functions and pathways, such as neurovisceral integration model (NIM) (Thayer and Lane, 2009) and polyvagal theory (Porges, 2018, 2022). However, one very interesting model of cardiac interoception is called the predictive coding model (or active inference), which suggests that the brain makes sense of multiple interacting streams of physiological interoceptive information by predicting their causes and generating prediction errors (Seth, 2013; Barrett and Simmons, 2015; Barrett et al., 2016; Seth and Friston, 2016; Stephan et al., 2016; Owens et al., 2018). This idea of psychopathology as a result of interoceptive error has been further developed by predictive coding frameworks. For example, it has been used to explain major depressive disorder (MDD) (Kube et al., 2020), anxious hypervigilance (Cornwell et al., 2017), but also autism (van Laarhoven et al., 2019) and altered conscious states, such as explaining a placebo induced analgesia (Büchel et al., 2014).

In the case of MDD (Kube et al., 2020), predictive coding assumes that an individual does not perceive the world as it actually is but through a lens which corresponds to the brains best guess of it (Ongaro and Kaptchuk, 2019). The brain handles uncertainty (entropy) of new events and environments by integrating information and making rational use of it, through applying prior knowledge in the interpretation of new observations (O'Reilly et al., 2012). In this case, their perception of the world corresponds to a negative perception which is consistent with their predictive (predictive coding) beliefs relating to their prior knowledge about the world (Rief and Joormann, 2019). This can include reinforced and derived failures that they have experienced in the world, and corresponding derived feelings of failure that they have made about themselves (their own concept of themselves). So, this skewed pattern of predictions may explain why people with MDD perceive the world in a predominantly negative way, thus creating a self-reinforcing negative feedback loop (Kube et al., 2020).

Specifically, in relation to ASD, Pellicano and Burr's (2012) general Bayesian account suggests that attenuated Bayesian priors (called hypo-priors) may be responsible for the unique perceptual experience of individuals with ASD. It is assumed that as perceptual experience is shaped by the integration of stimulus information both incoming interoceptive and other sensory information and prior background information about the world, the ASD individuals' perception is modulated to perceive the world more biased through sensory information (i.e., in a more real way) and largely ignores prior information. This modulation of perception through sensory experience favors local (real world) perception over global (knowledge-based) perception. This view is consistent with the theories of over selectivity, which assume that autism results from an overly strong perceptual focus on certain local features and which ignore contextual and other global characteristics (Edwards et al., 2012).

In predictive coding higher brain areas, attempt to predict and explain lower brain areas and project these predictions down to lower levels where the predicted information is subtracted (discounted) from the input (environmental or bodily) sensory information. This feedback of predictions to lower brain levels represents the empirically driven prior contextual background knowledge (Feldman and Friston, 2010). Conditions such as alexithymia could be related to high predictive errors, which could affect the shaping of a sense of embodied self (Seth and Friston, 2016), which may explain why individuals with alexithymia struggle to feel embodied emotions relating to themselves.

In relation to OCD, one review (Levy, 2018) suggested that in OCD, predictive coding errors were more weighted in a way (in the brain) that meant they were more costly for them than for healthy individuals. It is assumed that this added cost results in an upregulation of attention, which in turn leads to an overestimation of possible threats in the environment. Such an increase in perceived threat has been directly observed (Hezel and McNally, 2016).

Concepts such as selfhood are also important in therapeutic models, such as acceptance and commitment therapy (ACT) (Hayes et al., 2011; Hayes, 2019), a third wave behavioral therapy, which attempts to build psychological flexibility through six key processes: (1) present moment awareness; (2) openness and acceptance of painful feelings, thoughts, and memories; (3) cognitive defusion, the act of recognizing that thoughts are just thoughts and not to buy into them; (4) values orientation through identifying what is meaningful in your life; (5) commitment to values; (6) self-as-context (SAC), which is the awareness of thoughts and feelings but being an observer to these experience, i.e., the complete detachment of “self” from the literal meaning of thoughts.

Perhaps, the most relevant of these in relation to interception and selfhood is SAC (Zettle et al., 2018). In the ACT model, SAC relates to a “self” that once psychologically flexible is assumed not be a fixed state and instead a flexible and context dependent, as an observer to experience (i.e., the perspective-taking self) (McHugh and Stewart, 2012; McHugh et al., 2019). Some recent works have been conducted in the area of alexithymia, for instance through exploring the link between alexithymia and ISen, and within the context of mental health, psychological flexibility, and level of SAC (Edwards and Lowe, 2021). This found that SAC mediated the association between some of the ISen subconstructs and mental health, so clearly it is linked. This also means that it is potentially linked to the predictive coding account, whereby lower SAC, and perspective-taking skills likely means greater predictive errors.

Given the following problems: (1) the lack of clarity from the DSM which assumes latent syndromes should form discrete categories and cannot explain why there is co-morbidity between alexithymia, OCD, and autism; and (2) given that there seems to be a set of complex interactions between alexithymia, OCD, and autism in combination with SAC, psychological flexibility, and mental health, this study will seek to explore these in more detail. This study will therefore explore the complex causal sequences among ISen (interoception), mental health, psychological inflexibility, SAC, and how these relate to the conditions of OCD, alexithymia, and ASD.

This will be done through constructing a conceptual model which will explore direct and indirect sequential causality through a structural equation modeling (SEM). Previous studies have largely explored associations with the use of standard regression modeling and related statistical approaches (such as analysis of variance, ANOVA). However, regression analysis is not well-suited for describing causal sequences where there are potentially complex interactions between the variables. Many variables can be added in a multiple regression model, which can make it more comprehensive, but it still does not show causal sequences. Path models such as SEM allow the chains of conditional relations to be explore; therefore, it is ideal for representing causal models and it allows for both confirmatory and exploratory modeling.

However, before a conceptual model is constructed, it needs to be developed in stages. Questions that must be asked are (1) what potential confounds should be controlled for? (2) What should the structure of the conceptual model look like? (3) Which of the variables should mediate, if any? To answer these, standard statistical approaches (ANCOVA, regression, and simple mediation will be used) given that the literature provides less clues as to what the overall structure of such a conceptual model which captures such complex causal sequences should look like. For this approach, there will be four main stages in informing the development of a conceptual model and SEM of direct and indirect causality between these variables, and these include the following:

(1) Exploring potential confounds to a conceptual model through exploring how age effects vagal functioning when controlling for body mass index (BMI), gender, and comparing HRV at different age groups. Though there is evidence that age does affect vagal functioning is affected by age (Zhang, 2007), this phase will explore this while controlling for gender and BMI. This will lay the foundation for demonstrating how vagal function (which ISen interoception is based on) may deteriorate as a result of natural aging (it is hypothesized that participants in the older groups will have lower HRV than the younger groups). This will, therefore, provide useful knowledge about whether this should be controlled for in the SEM.

(2) Backwards stepwise regression models will explore which variables are most predictive of alexithymia, OCD, and autism to inform stage 3. It is hypothesized that all subconstructs and total values of ISen, alexithymia, OCD, alexithymia, and age would predict autism; all subconstructs and total values of mental health, ISen, alexithymia, autism, psychological flexibility, and age would predict OCD; and all subconstructs and total of values mental health, ISen, autism, and OCD would predict alexithymia. SAC is not hypothesized to directly predict these outcomes as will more likely act as an indirect effect.

(3) Five independent simple mediation analyses will be conducted, whereby some of the outcomes in the final regression models (of stage two) will inform the mediation analyses to be made. The mediation analyses will provide the conceptual model with the details of what structure the conceptual model and SEM should take through both direct and indirect effects. It is hypothesized that ISen self-regulation will mediate the association between OCD and autism, as well as alexithymia and depression. SAC will mediate the association between autism and alexithymia. Psychological inflexibility will mediate the association between ISen self-regulation and SAC, as well as SAC and ISen trusting.

(4) Finally, exploratory factor analysis and confirmatory factor analysis will demonstrate which item variables are the best fit for each loading of a latent variable in the SEM. This will be followed by the direct model fit test of the SEM to confirm the conceptual model. Tentative hypotheses for the SEM include the following: there will be a positive association between alexithymia and psychological inflexibility, a negative association between psychological inflexibility and SAC as well as ISen self-regulation, but a positive association with depression. There will be a negative association between ISen self-regulation and autism. There will be no direct effects between alexithymia and autism or depression, or OCD autism or depression, but there will be indirect effects of these *via* psychological inflexibility and SAC.

## Methods

### Participants

For the questionnaire, the inclusion criteria for participation were listed as follows: (1) participants needed to be at least 18 years of age; (2) have good ability to read English; (3) have normal to corrected to normal vision; (4) have internet access; and (5) and needed to report that they had received a diagnosis of autism from a healthcare professional. Participants were excluded if they did not meet these inclusion criteria requirements. For the ECG data, participants must have been 18 years, otherwise healthy with no specific diagnosis (refer to PhysioNet database for full details^1^.

In the first part of the study, 1,121 healthy participants were involved in ECG recordings, which measured healthy resting-state HRV and BMI. However, once missing data were removed (such as age), then 1,089 remained. In total, 242 participants took part in following survey and 18 withdrew without completing it, leaving 224 complete surveys recorded.

### Materials

The ECG data^2^ (Schumann and Bär, 2022) held on a publicly accessible database (PhysioNet) (Goldberger et al., 2000) were used for the baseline restful age-related cardiac change in HRV. Recordings were made through an ECG (lead II) at 1,000 Hz either by an MP150 (ECG100C, BIOPAC system inc., Golata, CA, USA) or Task Force Monitor system (CNSystems Medizintechnik GmbH, Graz AUT). These used pre-gelled Ag/AgCl electrodes (BlueSensor VL, Ambu BmbH, Bad Nauheim, GER) were attached according to an Einthoven triangle.

The survey section of data collection was conducted through Qualtrics^3^, which was distributed on various autism support groups on Facebook. Table 1 shows a scoring index for the questionnaires used (i.e., what a high or low score indicates). The survey included demographic questions about age, gender, and the following seven questionnaires:

**Table 1:** Index of measurement score meaning.

**Outcome measure/variable**	**Meaning**
AQ-10	Higher score indicates higher autism. Scoring sheet suggest referring yourself for a specialist diagnostic if the individual scores are >6 out of 10.
TAS-20	Higher score indicates higher alexithymia for the total. ≥ 61 = high alexithymia; ≤ 51 = low alexithymia (no alexithymia)
Y-BOCS-10	Higher score indicates higher OCD. Total score; 8–15 = Mild OCD; 16–23 = Moderate OCD; 24–31= Severe OCD; 32–40 = Extreme OCD
DAS-21	Higher scores indicates higher depression, anxiety, stress for the (three) relevant respective subsections.
AAQ-2	Higher score indicates higher inflexibility
SAC	Higher score indicates greater self-as-context
MAIA-2	Higher score indicates higher interoceptive awareness for the (eight) relevant respective subsections.

#### Autism spectrum quotient (AQ-10)

This is a 10 item scale and is a brief “red flag” assessment tool to help health professionals make a decision as to whether to make a referral for a full diagnostic assessment (Allison et al., 2012). The National Institute for Health Care Excellence (NICE, 2014) does not recommend any specific screening tool for adults and children with suspected moderate to severe autism; however, the AQ-10 is recommended in cases where there is not moderate to serve intellectual disabilities. This scale has four options to choose from “definitely agree” to “definitely disagree.” As a threshold of score of 6 for adults, sensitivity was 0.88, specificity was 0.91, and positive predictive value (PPV) was 0.85. Cronbach's alpha is also high on all measures (age groups) (>0.85) (Allison et al., 2012).

#### Yale-Brown obsessive compulsive scale (Y-BOCS)

This is a 10 item scale of OCD severity (Goodman et al., 1989) and is considered the gold standard for this measure and symptom severity (Storch et al., 2010). Participants can choose between five options, from 0 to 4 with varying answers, and include question such as “how much of your time is occupied by obsessive thoughts?” and “how much distress do your obsessive thoughts cause you?” It has five overall dimensions which questions are focused on: (1) time spent; (2) interference with functioning or relationships; (3) degree of distress; (4) resistance; and (5) control. Internal consistency of this measure is high, with a Cronbach's alpha of 0.89 and an interclass correlation >0.85 (Goodman et al., 1989).

#### Toronto alexithymia scale (TAS-20)

This is a 20 item scale of alexithymia, which specifically measures three constructs in addition to a total alexithymia score: (1) difficulty identifying feelings (DIF); (2) difficulty describing feelings (DDF); and (3) externally orientated thinking (EOT) (Bagby et al., 1994). Participants respond based on a five-point Likert scale, ranging from 1 = strongly disagree to 5 = strongly agree. The three subscales can also be combined to give a total alexithymia score, whereby scores of >61 indicate the presence of alexithymia, 52 to 60 indicate possible alexithymia, ≤ 51 indicate the absence of alexithymia. Construct validity of the scale (Pinaquy et al., 2003; Larsen et al., 2006; Pike, 2013) has been found to be high for DIF (Cronbach's alpha = 0.83) and DDF (Cronbach's alpha = 0.80), but not as high for EOT (Cronbach's alpha = 0.55).

#### Multi-dimensional assessment of interoceptive awareness (second version, MAIA-2)

This is a 32 item scale, with eight constructs (Mehling et al., 2018) of ISen (and not IAc). These constructs include the following: (1) noticing (noticing uncomfortable sensations in your body); (2) non-distracting (not ignoring painful bodily sensations); (3) not worrying (not worrying about bodily discomfort); (4) attention regulation (despite distractions, having the ability to place attention to one's body); (5) emotional awareness (noticing changes in one's body when your emotions change); (6) self-regulation (when one is feeling overwhelmed, being able to find a place of clam); (7) body listening (listening to one's body for emotional states); and (8) trusting (feeling at home in your body). These construct scales are rated from 0 to 5, whereby 0 = never and 5 = always. Higher scores indicate higher ISen for a particular subscale. Cronbach's alpha for all eight constructs ranged between 0.64 and 0.83. Only “noticing” (0.64) and “not worrying” (0.67) fell below the standard criterion of 0.7, indicating good overall construct validity.

#### The depression anxiety stress scales-21 (DAS-21, short-form)

This is a short version, which measures general ongoing (over the past week) psychological distress on three mental health subscales (anxiety, depression, and stress). The participant must enter a value between 0 and 3, which answers the degree to which a particular statement applied to them (0 = “did not apply to me at all” and 3 = “applied to me very much or most of the time”). Higher scores indicate higher levels of stress, anxiety, and depression. The measure has good construct validity, with a confirmatory factor analysis of 0.94. The measure also has good internal reliability as measured through Cronbach's alpha coefficients, which are 0.88 for depression, 0.82 for anxiety, 0.90 for stress, and 0.93 for the total scale (Henry and Crawford, 2005).

#### Acceptance and action questionnaire (second version; AAQ-2)

This is a seven item scale, which measures general psychological inflexibility (Bond et al., 2011). The questions ask the participant how they accept and open up to difficult thoughts and feelings, as well as how they engage in valued behavior when difficult thoughts and feelings are present. The participant must enter a value between 1 and 7, whereby 1 = never true and 7 = always true. Higher scores indicate higher psychological inflexibility. The measure has good construct validity with a Cronbach's alpha of 0.83 (Frewen et al., 2008).

#### Self-as-context (SAC) scale

This is an 11 item self-report scale, where participants must respond on a seven point Likert scale with responses ranging from 1= strongly agree to 7 = strongly disagree, where higher scores indicate higher self-as-context (Gird, 2013). The item statements include, for example, “I have a perspective on life that allows me to deal with life's disappointments without getting overwhelmed by them.” and “Even though there have been many changes in my life, I'm aware of a part of me that has witnessed it all.” The scale has good construct validity with a Cronbach's alpha of 0.82.

### Procedure

For the ECG data, refer to their website^4^ for full details, but the key points are summarized here. Participants were instructed to lie down on an examination tilt table in a temperature-controlled room of 22°C. During the recordings, it was quiet and fully shaded. For resting-state recordings, participants were instructed to avoid movement and the instructor waited a few minutes for the participants to relax while checking the quality of the signals (electrodes were repositioned if the signal was noisy). The length of the recordings averaged 19 min and was supervised at all times by the instructor.

For the questionnaire, an advertisement was placed on autism support groups on Facebook, which explained the study in some details and provided an email that interested individuals could respond to. Once participants responded to the advertisement, they were then given a Qualtrics link that contained full information about the study, ethical implications, and a consent form. On consent, participants were then provided with seven questionnaires (refer to Materials section) in addition to demographic questions *via* Qualtrics.

### Ethical statement

Ethics were approved through Swansea University's Department of Psychology Research Ethics Council (REC). This was in accordance with the Declaration of Helsinki and included obtaining written informed consent from all participants, right to withdraw, and a full debriefing at the end of the study.

### Data analysis

For the first phase of the study, ECG data^5^ (Schumann and Bär, 2022) held on a publicly accessible database (PhysioNet) were analyzed using the Matlab PhysioNet tool, cardiovascular analysis toolbox to produce the HRV analysis, which included artifact removal of the data. IBM's SPSS, version 28.0. was used to conduct a general linear model, analysis of covariance (ANCOVA). Here, the data age groups were collapsed into three groups as follows: group one included ages 18 to 29, group two included ages 30 to 44, and group three included ages 45 and over. Gender and BMI were included as covariates in this model.

For the second phase of the study questionnaires, there were no missing data as the participants were required to complete all questions. For this, a rule was set in Qualtrics, which prevented participants from progression through the questionnaire without first completing the questions on each page (i.e., a reminder appeared if they missed a question), as in previous studies (Edwards, 2019; Edwards and Lowe, 2021).

Descriptive and inferential statistics (regression and correlations) were carried out using IBM's SPSS, version 28.0. As the associations among alexithymia, autism, and OCD are largely uncertain, assumptions about a predefined hierarchy as required for hierarchical regression models could not be achieved. So, instead, stepwise backwards regressions were chosen over a hierarchical regression as the hypothesis assumptions are much more tentative. This included 19 predictors: age, the two sub measures Y-BOCS (OCD), the three sub measures TAS-20 (alexithymia), the three sub measures of DAS-21 (mental health); AAQII (psychological inflexibility), SAC, the eight sub measures of MAIA-2 (IAw), and the dependent measure of autism (AQ10).

For the stepwise backwards regressions, the selection criteria determine which of the variables are retained at each step. This selection criteria only retains variables in each step if they improve the overall goodness of model fit as indexed by *R*^2^, and minimize the complexity of the model as indexed by the Akaike information criterion (AIC) which is based on information theory) (Akaike, 1974).

A power calculation utilizing G^*^Power^6^ version 3.1.9.7 (Faul et al., 2007) was conducted that determined whether there was sufficient power to input the 19 predictors into the stepwise backwards regression. For 19 predictors, with power set to 0.8, and a medium effect size specified by Cohen's criteria of 0.15 *f* ^2^ (Cohen, 1988) an a priori assumption of 153 participants was calculated. A larger sample size of 224 participants (who completed the survey), was obtained, whereby a *post hoc* analysis, assuming a medium effect size, calculated power of 0.96. Given this power, there is only 3% chance of making a type two error (failing to reject the null hypothesis when it should have been) (Cohen, 1988).

In addition to the regression analyses, as part of phase three of the study, further mediation analyses were conducted using the SPSS PROCESS macro (model 7) by Andrew Hayes (Hayes, 2013). Mediation (or indirect effects) is the effect of independent variable *X*_1_ on dependent variable *Y* goes through a mediator *X*_2._ This is commonly defined as the reduction of the regression coefficient of *X*_1_ on *Y*, when *X*_2_ is controlled for (Judd and Kenny, 1981; Baron and Kenny, 1986). Baron and Kenny (Baron and Kenny, 1986) proposed a four step approach for simple mediation, whereby all of these steps need to be significant. The first step is to conduct a simple regression with *X* predicting *Y* to test the coefficient of path *c*, expressed as, *Y* = *B*_0+_ *B*_1_ *X* + *e*. The second step is to conduct a simple regression with *X* predicting *M* (the mediator), called path a, and expressed as *M* = *B*_0+_ *B*_1_ *X* + *e*. The third step is to conduct a regression with *M* predicting *Y*, called the *b* path and expressed as *Y* = *B*_0+_ *B*_1_ *M* + *e*. The final step is to conduct a regression whereby *X* and *M* predict *Y* (*X* controlled for, or mediated by *M*) and expressed as *Y* = *B*_0+_ *B*_1_ *X*+*B*_2_ *M* + *e*. Once mediated, if *X* no longer predicts *Y*, this is a full mediation (i.e., path *c'*), and if not, then this is instead considered a partial mediation (as long as the difference is significant between *c* and *c'*, i.e., confidence intervals do not cross zero).

The regression outcomes, along with the literature, helped to inform the development of a conceptual model, which could be tested through an SEM. Regression analysis is not well-suited for describing causal sequences. Path models such as SEM allow the chains of conditional relations to be explored, and this includes mediation hypotheses, so helps with evaluating causal hypotheses. For the SEM of part of the study, IBM's AMOS version 28.0. was used.

## Results

### Baseline aging effects of the vagus nerve HRV functioning in healthy controls

Aging of the vagal nerve that modulates the sympathetic and parasympathetic functioning of the ANS was explored. Table 2 shows the descriptive statistics, whereby HRV is higher for the youngest age group (M = 50.84, SD = 25.05), followed by the second youngest age group (M = 39.72, SD = 28.65), and lowest for the oldest age group (M = 30.99, SD = 33.57). One-way analysis of covariance (ANCOVA) was utilized to explore the HRV differences across the four age groups (the data were compressed into four groups) whereas gender and BMI were included as control covariates. A Shapiro–Wilk test revealed the data to be non-normal; however, when sample size is >40, the violation to this assumption does not cause a problem (Pallant, 2020). Furthermore, in cases where there are hundreds of participants (as is the case with this sample), the distributions of data can be ignored (Altman and Bland, 1995) and the parametric test can be used (Elliott and Woodward, 2007). HRV differed according to BMI [*F*_(1, 1087)_ = 5.56, *p* < 0.05, $\eta_{p}^{2}$ = 0.005] and age [*F*_(2, 1087)_ = 11.89, *p* < 0.001, $\eta_{p}^{2}$ = 0.021], but not gender [*F*_(1, 1087)_ = 1.23, *p* = 0.27, $\eta_{p}^{2}$ = 0.001]. Most importantly, age when controlling for gender and BMI was still highly significant [*F*_(2, 1090)_ = 26.41, *p* < 0.001, $\eta_{p}^{2}$ = 0.046]. *Post hoc* Bonferroni adjusted tests indicated that HRV differences between all age groups were significant (*p* < 0.001).

**Table 2:** Descriptive statistics.

**Variable**	**Mean (SD)**
Age group 1 (18 to 29)	50.84 (25.05)
Age group 2 (30 to 44)	39.72 (28.65)
Age group 3 (45 +)	30.99 (33.57)

### Questionnaire data of clinical sample

Table 3 shows the descriptive statistics, where mean ASQ-10 score was 6.87 (SD = 2.37) and this was within the range for suspected autism whereby the questionnaire criteria suggest that a referral to a specialist should be made. Y-BOCS-10 mean score for OCD was 16.71 (SD = 6.91) and this was within the range for moderate OCD. TAS-20 mean score was 63.34 (SD = 11.04), and this was in the range for high alexithymia. The skewness and kurtosis were all within a value of plus or minus two, indicating a normal distribution for each variable (George and Mallery, 1999). On inspection of the heteroskedasticity visual plot of the residuals, these were observed to be homoscedastic. Before regression analysis was conducted, preliminary analyses were conducted to ensure that there were no violations to the assumptions of multi-collinearity, normality, linearity, and homoscedasticity. The correlations showed that the predictors were below 0.8 (refer to Table 4), with the VIF scores of the coefficients below 10, indicating that these were free from multi-collinearity.

**Table 3:** Descriptive statistics and normality scores of the variables.

**Variable**	**Mean (SD)**	**Minimum–maximum**	**Skewness**	**Kurtosis**
Age	31.61 (10.16)	18–73	1.14	1.34
ASQ-10	6.87 (2.37)	1–10	−0.58	−0.56
Y-BOCS	16.71 (6.91)	0–38	0.16	0.17
Y-BOCS Obsession	8.85 (3.61)	0–19	0.42	0.17
Y-BOCS Compulsion	7.85 (3.90)	0–19	0.43	−0.04
AAQ-2	33.56 (8.43)	7–49	−0.41	0.30
SAC	46.71 (12.06)	17–77	−0.20	−0.14
DAS-21 Stress	27.06 (10.87)	0–48	−0.28	−0.40
DAS-21 Anxiety	19.64 (10.09)	0–42	0.06	−0.70
DAS-21 Depression	23.43 (11.15)	0–42	−0.15	−0.92
TAS-20 Total	63.34 (11.04)	30–89	−0.34	−0.13
TAS-20 DIF	23.96 (5.98)	7–35	−0.51	−0.39
TAS-20 DDF	18.15 (4.17)	5–25	−0.78	0.37
TAS-20 EOT	21.23 (4.27)	11–33	−0.07	−0.19
MAIA-2 Not-noticing	2.89 (1.07)	0–5	−0.02	−0.39
MAIA-2 Not-distracting	2.12 (1.01)	0–4	0.02	−0.07
MAIA-2 Not worrying	2.04 (0.99)	0–5	−0.03	−0.43
MAIA-2 Attention regulation	1.97 (0.97)	0–5	0.40	0.04
MAIA-2 Emotional awareness	2.79 (1.09)	0–5	0.01	−0.31
MAIA-2 Self-regulation	1.83 (1.14)	0–5	0.46	−0.29
MAIA-2 Body listening	1.80 (1.18)	0–5	0.47	−0.19
MAIA-2 Trusting	2.10 (1.39)	0–5	0.39	−0.65

**Table 4:** Correlations between variables.

	**1**	**2**	**3**	**4**	**5**	**6**	**7**	**8**	**9**	**10**	**11**	**12**	**13**	**14**	**15**	**16**	**17**	**18**	**19**	**20**	**21**
2	0.27**																				
3	0.08	0.24**																			
4	0.10	0.21**	0.92**																		
5	0.05	0.24**	0.93**	0.69**																	
6	−0.04	0.26**	0.47**	0.52**	0.36**																
7	0.07	−0.26**	−0.25**	−0.29**	−0.17**	-.51**															
8	0.04	0.29**	0.50**	−0.48**	−0.45**	0.61**	−0.34**														
9	−0.15*	0.13	0.56**	0.53**	0.51**	0.51**	−0.28**	0.70**													
10	−0.07	0.23**	0.41**	0.42**	0.34**	0.68**	−0.46**	0.72**	0.54**												
11	0.09	0.40**	0.29**	0.29**	0.25**	0.41**	−0.38**	0.44**	0.34**	0.36**											
12	0.06	0.39**	0.37**	0.39**	0.29**	0.50**	−0.37**	0.54**	0.46**	0.43**	0.86**										
13	0.08	0.35**	0.19**	0.18**	0.17*	0.29**	−0.14*	0.33**	0.24**	0.19**	0.81**	0.62**									
14	0.09	0.16*	0.05	0.01	0.08	0.07	−0.33**	0.07	0.01	0.13	0.59**	0.20**	0.26**								
15	−0.23**	−0.24**	0.19**	0.15*	0.19**	0.02	0.17*	0.09	0.29**	0.06	−0.13*	−0.06	−0.09	−0.16*							
16	0.46	−0.01	−0.04	−0.87	0.01	−0.14*	−0.01	−0.13	−0.17*	−0.17*	−0.26**	−0.22**	−0.24**	−0.12	0.02						
17	0.11	−0.07	−0.24**	−0.23**	−0.21**	−0.27**	−0.21**	−0.38**	−0.33**	−0.25**	−0.21**	−0.31**	−0.17*	0.06	−0.13*	−0.05*					
18	−0.22*	−0.45**	−0.19**	−0.22**	−0.15*	−0.29**	0.46**	−0.28**	−0.09	−0.25**	−0.44**	−0.42*	−0.36**	−0.19*	0.44**	−0.06	0.19**				
19	−0.16**	−0.21**	0.11	0.12	0.07	−0.01	0.27**	0.05	0.19**	−0.05	−0.08	0.02	−0.06	−0.18**	0.62**	−0.03	−0.24**	0.42**			
20	−0.19**	−0.38**	−0.22**	−0.22**	−0.19**	−0.29**	0.41**	−0.26**	−0.03	−0.26**	−0.27**	−0.25**	−0.14**	−0.21**	0.36**	−0.09	0.07	0.60**	0.50**		
21	−0.11	−0.33**	−0.07	−0.08	−0.04	−0.17**	0.37**	−0.09	0.01	−0.23**	−0.25**	−0.16*	−0.21**	−0.22**	0.39**	0.05	−0.06	0.54**	0.58**	0.61**	
22	−0.15*	−0.37**	−0.20**	−0.25**	−0.12	−0.46**	0.62**	−0.34**	−0.20**	−0.44**	−0.50**	−0.49**	−0.36**	−0.25**	0.35**	0.11	0.18**	0.60**	0.43**	0.57**	0.53**

1. Age; 2. AQ10; 3. Y-BOCS Total; 4. Y-BOCS Obsession; 5. Y-BOCS Compulsion; 6. AAQII; 7. SAC; 8. DAS-21 Stress; 9. DAS-21 Anxiety; 10. DAS-21 Depression; 11. TAS-20 total; 12. TAS-20 DIF; 13. TAS-20 DDF; 14. TAS-20 EOT; 15. MAIA-2 Not-noticing; 16. MAIA-2 Not-distracting; 17. MAIA-2 Not worrying; 18. MAIA-2 Attention regulation; 19. MAIA-2 Emotional awareness; 20. MAIA-2 Self-regulation; 21. MAIA-2 Body listening; 22. MAIA-2 Trusting. Sig, 2 tailed; *p <0.05; **p <0.01.

Table 4 shows the correlations between the variables. There was a positive correlation between age and autism, but a negative correlation between age and anxiety. Autism was positively correlated with stress and depression. Age was also negatively correlated with several of the ISen indices but did not correlate with either the alexithymia scale or OCD, suggesting that it is related specifically to autism (i.e., the older individuals were more likely to be autistic in this sample). SAC was negatively correlated with OCD, autism, and alexithymia scales. There was also a positive correlation among psychological inflexibility and OCD, autism, and alexithymia scales. Autism, OCD, and alexithymia all were negatively correlated with several of the ISen subscales, suggesting that their interceptive sensibility was low for these conditions. Autism, OCD, and alexithymia all positively correlated with several mental health (DAS-21) dimensions, suggesting that mental health issues were high in these conditions.

Tables 5–**7** show the outcomes of the three stepwise backwards regression models. Table 5 specifically shows all of the variables (subscales were chosen instead of total scores where they were available) entered as predictors and regressed against autism as the DV. Here, the adjusted *R*^2^ = 0.31, and the model was significant [*F*_(6, 217)_ = 17.51, *p* < 0.001]. In the final step of the model, age (standardized beta coefficients expressed here, β = 0.18), gender (β = 0.13), OCD compulsion (β = 0.14), alexithymia DFF (β = 0.19), ISen attention regulation (β = −0.21), and ISen self-regulation (β = −0.17) were all selected.

**Table 5:** Regression model summary where autism is the DV.

**Variable**	**Standardized β**	**S.E**.	***t*-value**	***P*-value**
Age	0.18	0.01	3.18	<0.01
Gender	0.13	0.21	2.29	<0.05
OCD Compulsion	0.14	0.04	2.48	<0.05
Alexithymia DDF	0.19	0.03	3.28	<0.01
ISen attention regulation	−0.21	0.18	−2.75	<0.01
ISen self-regulation	−0.17	0.15	−2.36	<0.05

For the second regression model (refer to Table 6), all of the variables (subscales were chosen instead of total scores where they were available) were entered into the regression model except for the two OCD subscales (obsession and compulsion), which were excluded as total OCD was the DV. Here, the adjusted *R*^2^ = 0.43, and the model was significant [*F*_(7, 216)_ = 24.61, *p* < 0.001]. In the final step of this model, age (β = 0.12), gender (β = −0.15), autism (β = 0.12), psychological inflexibility (β = 0.19), anxiety (β = 0.42), ISen not noticing (β = 0.18), and ISen self-regulation (β = −0.46) were all selected.

**Table 6:** Regression model summary where OCD is the DV.

**Variable**	**Standardized β**	**S.E**.	***t*-value**	***P*-value**
Age	0.12	0.04	2.10	<0.05
Gender	−0.15	0.57	−2.94	<0.01
Autism	0.12	0.17	1.96	=0.05
Psychological inflexibility	0.19	0.05	2.97	<0.01
Anxiety	0.42	0.04	6.69	<0.001
ISen self-regulation	−0.46	0.38	3.05	<0.01
ISen not noticing	0.18	0.36	−2.44	<0.05

For the third regression model (refer to Table 7), all of the variables (subscales were chosen instead of total scores where they were available) were entered into the regression model except for the three alexithymia subscales; DIF, DDF, and EOT which were excluded, whereby the DV was this time specified as total alexithymia. Here, the adjusted *R*^2^ = 0.42, and the model was significant [*F*_(6, 217)_ = 27.49, *p* < 0.001]. In the final step of this model, autism (β = 0.17), stress (β = 0.21), ISen not distracting (β = −0.20), ISen attention regulation (β = −0.20), ISen emotion awareness (β = 0.15), and ISen trusting (β = −0.29) were selected.

**Table 7:** Regression model summary where alexithymia is the DV.

**Variable**	**Standardized β**	**S.E**.	***t*-value**	***P*-value**
Autism	0.17	0.27	2.89	<0.01
Stress	0.21	0.06	3.54	<0.01
ISen not distracting	−0.20	0.57	−3.87	<0.01
ISen attention regulation	−0.20	0.79	−2.86	<0.01
ISen emotion awareness	0.15	0.61	2.44	<0.05
ISen trusting	−0.29	0.55	−4.13	<0.01

### Mediation models

**Figure 1:**
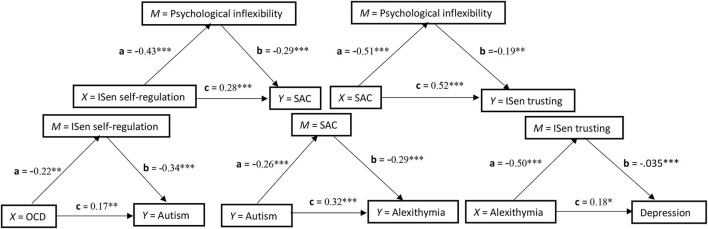
Five simple independent mediation models stacked in conceptual hierarchy and showing the standardized coefficients of each path. *p < 0.05; **p < 0.01; ***p < 0.001.

A number of five separate mediation models were conducted, and these are displayed in Figure 1 and stacked in a conceptual hierarchy. The structure of the mediation models was informed by both the literature and also the regression models performed previously. In the first, OCD (*X*) was explored as a predictor for autism (*Y*), and ISen self-regulation was chosen as a mediator (*M*). The direct association between OCD (*X*) and autism (*Y*) was significant [*c* = *t*_(221)_ = 2.66, *p* < 0.01, β = 0.17]. The direct association between OCD (*X*) and the mediator (*M*) ISen self-regulation was also significant [*a* = *t*_(222)_ = −3.35, *p* < 0.01, β = −0.22]. The direct association between the mediator (*M*) ISen self-regulation and autism (*Y*) was significant [*b* = *t*_(221)_ = −5.46, *p* < 0.001, β = −0.34]. To indicate a mediation effect, the indirect association of *X* to *Y via M* was significant as confidence intervals at 95% did not cross zero [CI = 0.01, 0.05]. However, as the variance of the direct path of *X* to *Y* did not reduce to zero after the mediator was added (i.e., the *c'* path), this indicated a partial mediating effect.

In the second mediation analysis, autism (*X*) was explored as a predictor for alexithymia (*Y*) and SAC was chosen as a mediator (*M*). The direct association between autism (*X*) and alexithymia (*Y*) was significant [*c* = *t*_(221)_ = 5.36, *p* < 0.001, β = 0.32]. The *a* path association between autism (*X*) and the mediator (*M*) SAC was also significant [*t*_(222)_ = −3.97, *p* < 0.001, β = −0.26)]. The *b* path association between the mediator (*M*) SAC and alexithymia (*Y*) was significant [*b* = *t*_(221)_ = −4.89, *p* < 0.001, β = −0.29]. To indicate a mediation effect, the indirect path of *X* to *Y via M* was significant as confidence intervals at 95% did not cross zero [CI = 0.14, 0.59]. However, as the variance of the direct path of *X* to *Y* did not reduce to zero after the mediator was added (i.e., the *c'* path), this indicated a partial mediating effect.

In the third mediation analysis, alexithymia (*X*) was explored as a predictor for depression (*Y*) and ISen trusting was chosen as a mediator (*M*). The direct association between alexithymia (*X*) and depression (*Y*) was significant [*c* = *t*_(221)_ = −5.15, *p* < 0.05, β = 0.18]. The *a* path association between alexithymia (*X*) and the mediator (*M*) ISen trusting was also significant [*t*_(222)_ = −8.67, *p* < 0.001, β = −0.50]. The *b* path association between the mediator (*M*) ISen trusting and depression (*Y*) was significant [*t*_(221)_ = −5.15, *p* < 0.001, β = −0.35]. To indicate a mediation effect, the indirect path of *X* to *Y via M* was significant as confidence intervals at 95% did not cross zero [CI = 0.09 to 0.28]. However, this was a partial mediation as the variance of the direct path of *X* to *Y* did not reduce to zero after the mediator was added (i.e., the *c'* path).

In the fourth mediation analysis, ISen self-regulation (*X*) was explored as a predictor for SAC (*Y*) and psychological inflexibility was chosen as a mediator (*M*). The direct association between ISen self-regulation (*X*) and SAC (*Y*) was significant [*c* = *t*_(221)_ = 4.94, *p* < 0.001, β = 0.28]. The *a* path association between ISen self-regulation (*X*) and the mediator (*M*) psychological inflexibility was also significant [*t*_(222)_ = −7.53, *p* < 0.001, β = −0.43]. The *b* path association between the mediator (*M*) psychological inflexibility and SAC (*Y*) was significant [*b* = *t*_(221)_ = −4.56, *p* < 0.001, β = −0.29]. To indicate a mediation effect, the indirect path of *X* to *Y via M* was significant as confidence intervals at 95% did not cross zero [CI = 0.69 to 2.13]. However, this was a partial mediation as the variance of the direct path of *X* to *Y* did not reduce to zero after the mediator was added (i.e., the *c'* path).

In the fifth mediation analysis, SAC (*X*) was explored as a predictor for ISen trusting (*Y*) and psychological inflexibility was chosen as a mediator (*M*). The direct association between SAC (*X*) and ISen trusting (*Y*) was significant [*c* = *t*_(221)_ = 8.65, *p* < 0.001, β = 0.52]. The *a* path association between SAC (*X*) and the mediator (*M*) psychological inflexibility was also significant [*a* = *t*_(222)_ = −8.92, *p* < 0.001, β = −0.51]. The *b* path association between the mediator (*M*) psychological inflexibility and ISen trusting (*Y*) was significant [*b* = *t*_(221)_ = −3.28, *p* < 0.01, β = −0.19]. To indicate a mediation effect, the indirect path of *X* to *Y via M* was significant as confidence intervals at 95% did not cross zero [CI = 0.004 to 0.02]. However, this was a partial mediation as the variance of the direct path of *X* to *Y* did not reduce to zero after the mediator was added (i.e., the *c'* path).

### SEM analysis

The first SEM was conducted to explore autism and mental health as two separate DVs. The first step in the process was to identify applicable latent variables. This was done first through exploratory factor analysis (EFA), whereby the Kaiser–Mayer–Olkin (KMO) measure of sampling adequacy (KMO = 0.81) exceeded the desired cutoff score of 0.70 (Kaiser, 1974), indicating the data represented high sampling adequacy. Bartlett's test of sphericity [$\chi_{\left(120\right)}^{2}$ = 1677.08, *p* < 0.001] was significant, indicating that the matrix was not an identity matrix, which means the variables are sufficiently related to one another, therefore, indicating that it is acceptable to use EFA. The communalities table which utilized maximum likelihood as the extraction method revealed that three items were smaller than 0.3, and these were EOT = 0.11, ISen not distracting = 0.09, and ISen not worrying = 0.19. Total variance loaded onto four factors, whereby the cumulative value of four factors equaled 54.79% of the variance, and goodness of fit was at an acceptable level and significant [$\chi_{\left(62\right)}^{2}$ = 158.01, *p* < 0.001].

The pattern matrix demonstrated that EOT, ISen not distracting, and ISen not worrying were removed from the factor loading (as their communality extraction values were below 0.3 and therefore deemed too low). There were no cross-loadings, and the pattern matrix is illustrated in Table 8. The factor correlation matrix indicated that no factor loadings were >0.7 and were, therefore, within an acceptable range.

**Table 8:** Pattern matrix of four groups as identified through EFA.

**Variable**	**Factor 1**	**Factor 2**	**Factor 3**	**Factor 4**
Obsession			0.77	
Compulsion			0.93	
DAS21-Stress		0.87		
DAS21-Anxiety		0.62		
DAS21-Depression		0.91		
DIF				0.80
DDF				0.78
ISen Not noticing	0.64			
ISen Attention regulation	0.65			
ISen Emotion awareness	0.81			
ISen Self-regulation	0.76			
ISen Bodily listening	0.77			
ISen Trusting	0.61			

Following the EFA, a confirmatory factor analysis (CFA) was conducted whereby the four variables groups created by EFA were converted into latent variables called mental health, alexithymia, OCD, and ISen. Covariance was then added between all latent variables and combinations (threshold for modification indices was set to 10 and the number of bootstrap samples was set to 1000). However, the initial CFA did not indicate a good model fit, root mean square error of approximation (RMSEA) was >0.1, and comparative fit index (CFI) and Tucker–Lewis index (TLI) were lower than 0.9. As a result of this, modification indices were checked and was the standardized residual covariance matrix table. Several ISen items were above a standardized residual covariance value of two and were removed with RMSEA, CFI, and TLI retested for fitness of model. Once high values were removed, only the item ISen self-regulation was maintained from the ISen latent variable and kept as a standalone direct measure of itself. “Anxiety” and “stress” were removed from the latent variable “mental health,” leaving depression as standalone direct measure of itself. All other items within the latent variables “alexithymia” and “OCD” were maintained. The final model fit indicated RMSEA = 0.30, CFI= 0.99, and TLI = 0.99 indicating an excellent fit. CFI and TLI values ≥0.95 and RMSEA values ≤ 0.08 indicate the data which are well fitting (Hu and Bentler, 1998, 1999). The acceptable range for the standardized root mean squared residual (SRMR) index is between 0 and 0.08 (Hu and Bentler, 1999).

**Figure 2:**
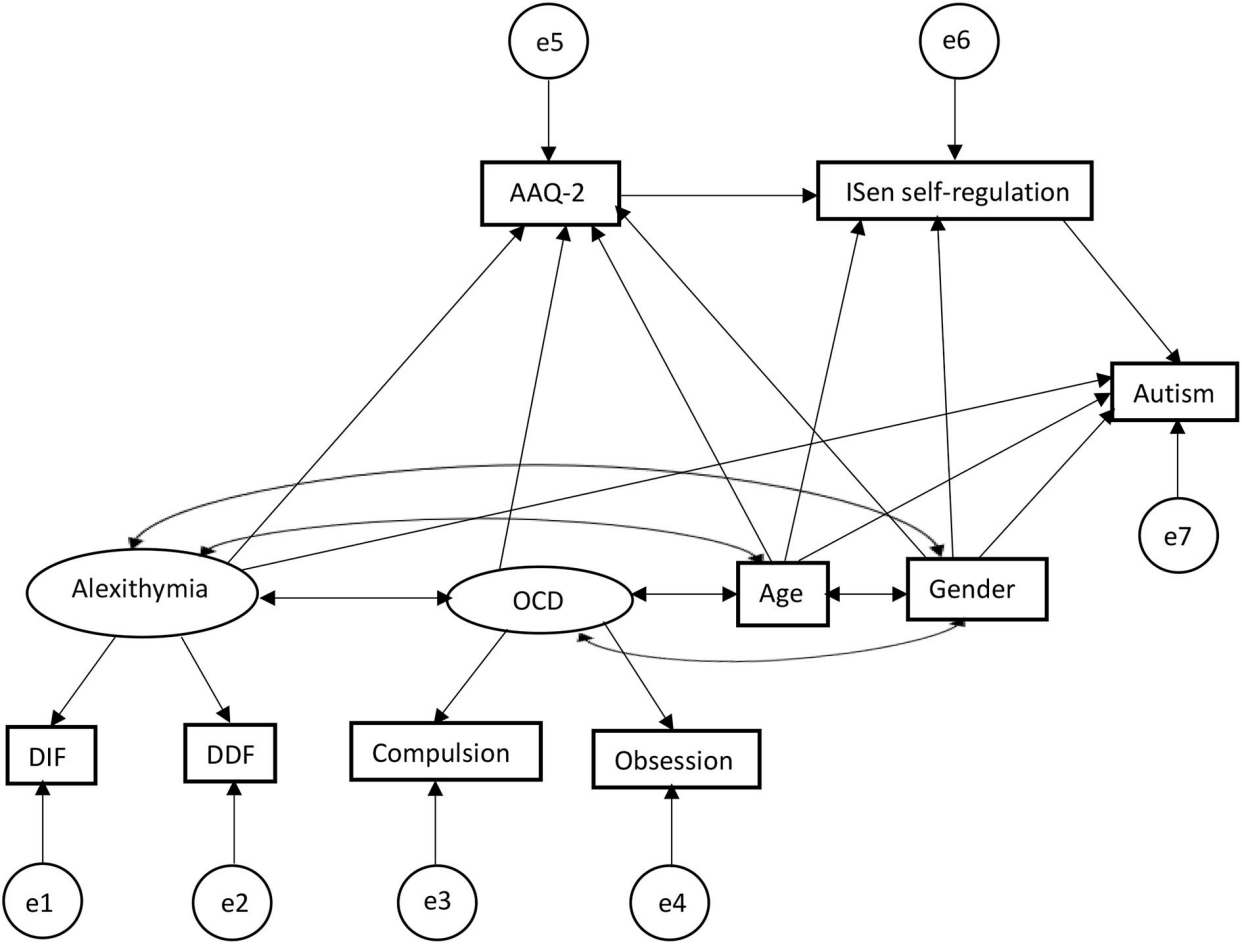
SEM first output predictors for autism, showing alexithymia and OCD are related indirectly (alexithymia *via* serial mediation) through psychological inflexibility and ISen self-regulation in predictively causing autism.

The remaining conceptual model was tested within a SEM (refer to Figure 2) (with 1000 bootstrap samples). SEM fits (through RMSEA, CFI, TLI, and SRMR) were explored. This model produced a model fit: [*X*^2^(*df* =13) = 15.19, *p* = 0.29], whereby this *X*^2^ test indicates the extent to which the model covariance matrix deviates from the sample covariance matrix and tests that deviation against a null hypothesis of zero (i.e., it is not significantly different) (Peugh and Feldon, 2020). So, a non-significant finding here indicates that the proposed model is a good fit of the data (it is not significantly different from the representation of the data). In addition to this, RMSEA = 0.03, comparative fit index (CFI) = 0.99; Tucker–Lewis index (TLI) = 0.99, GFI = 0.99, SRMR = 0.03. Alexithymia covaried with OCD (correlation values given, *r* = 0.39), with age (*r* = 0.06), with gender (*r* = 0.17); OCD covaried with age (*r* = 0.10), with gender (*r* = −0.09); and age covaried with gender (*r* = -0.11).

For direct effects, alexithymia was significantly positively associated with psychological inflexibility (reporting standardized beta coefficients) (β = 0.36, CI = [0.21, 0.52], *p* < 0.001); alexithymia was also significantly positively associated with autism (β = 0.27, CI = [0.10, 0.49], *p* < 0.001); alexithymia was not significantly associated with ISen self-regulation (β = −0.13, CI = [−0.31, 0.05], *p* = 0.11); OCD was significantly positively associated with psychological inflexibility (β = 0.39, CI = [0.24, 0.53], *p* < 0.001); psychological inflexibility was significantly negatively associated with ISen self-regulation (β = −0.24, CI = [−0.41, −0.07], *p* < 0.01). ISen self-regulation was significantly negatively associated with autism (β = −0.26, CI = [−0.39, −0.11], *p* < 0.001). For control measures, age was significantly negatively associated with ISen self-regulation (β = −0.19, CI = [−0.29, −0.07], *p* < 0.01]. Age was also significantly associated with autism (β = 0.22, CI = [0.12, 0.32], *p* < 0.001). However, age was not significantly associated with psychological inflexibility (β = −0.10, CI = [−0.19, −0.00], *p* = 0.06. Gender was not significantly associated with neither ISen self-regulation (β = 0.03, CI = [−0.02, 0.03], *p* = 0.89), autism (β = 0.11, CI = [0.00, 0.21], *p* = 0.05), nor psychological inflexibility (β = −0.01, CI = [−0.11, 0.09], *p* = 0.89).

In terms of mediating indirect effects, age was indirectly associated with ISen self-regulation *via* psychological inflexibility (β = 0.02, CI = [0.00, 0.06], *p* < 0.05); alexithymia was indirectly associated with ISen self-regulation *via* psychological inflexibility (β = −0.09, CI = [−0.19, −0.03], *p* < 0.01). OCD was indirectly associated with ISen self-regulation *via* psychological inflexibility (β = −0.09, CI = [−0.18, −0.03], *p* < 0.01). Psychological inflexibility was indirectly associated with autism *via* ISen self-regulation (β = 0.06, CI = [0.02, 0.15], *p* < 0.01).

**Figure 3:**
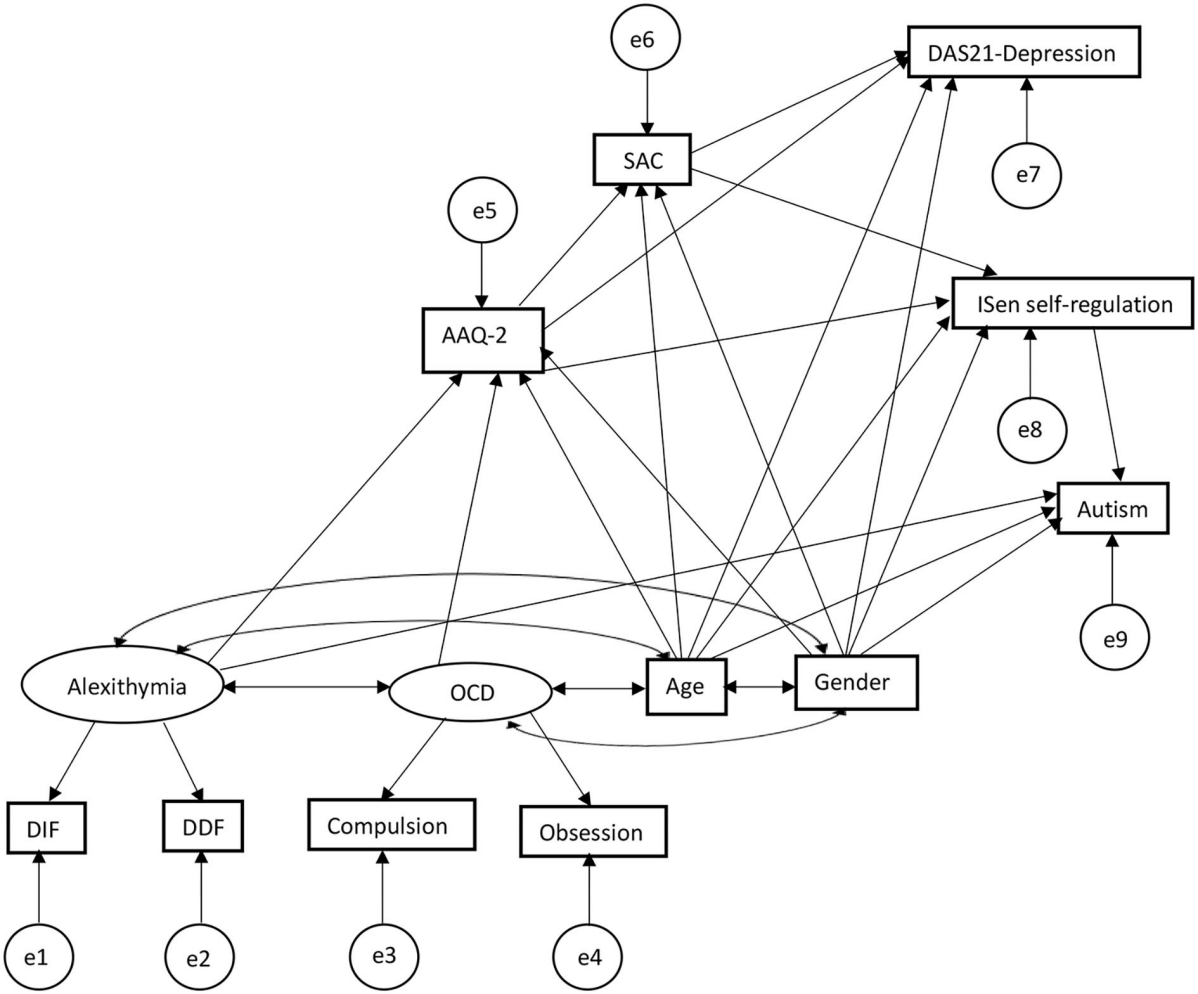
SEM second output extended the first SEM in Figure 2 adding SAC and depression scale, showing a second and third mediation of SAC mediating the relationship between AAQ-2 and ISen self-regulation, and the relation between AAQ-2 and DAS-21 depression.

In the second SEM (refer to Figure 3), depression was added as a second DV in addition to autism, which also included adding SAC into the model to account for this. This model produced again a good model fit: [*X*^2^(*df* = 24) = 34.75, *p* = 0.07], RMSEA = 0.04, CFI = 0.99; TLI = 0.97. GFI = 0.97, SRMR = 0.03. Some of the beta coefficients change as a result of the new variables added while others remained the same. Alexithymia covaried with OCD (correlational values given, *r* = 0.39), with age (*r* = 0.06), with gender (*r* = 0.17); OCD covaried with age (*r* = 0.10), with gender (*r* = −0.09); and age covaried with gender (*r* = −0.11).

**Figure 4:**
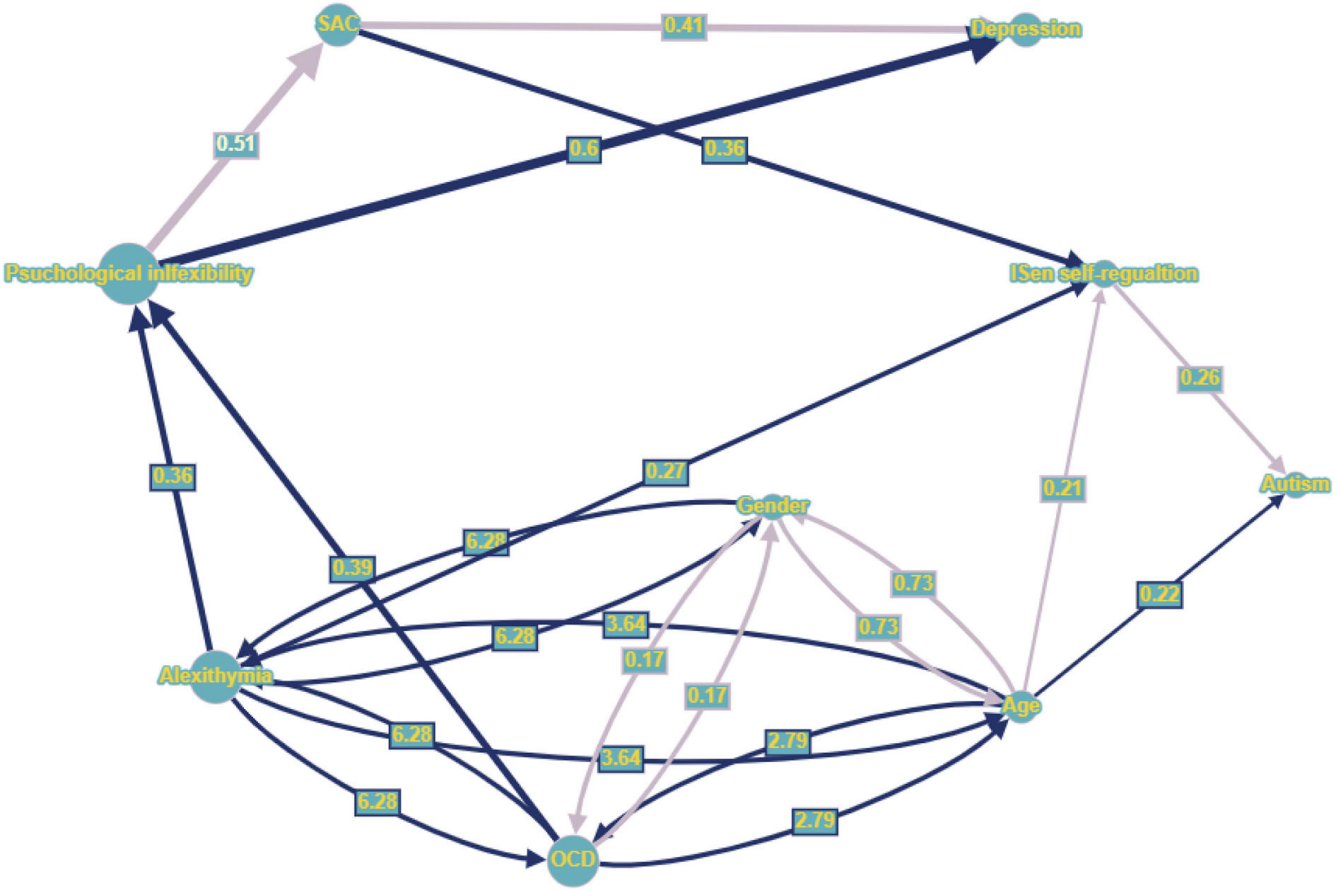
Graph nodes and edges representation (larger nodes and wider lines indicates those with greater effect within the network), whereby dark blue directed lines indicate positive standardized regression coefficients (only significant coefficients presented), light purple directed line represent negative regression coefficients. Dark blue bidirectional lines represent positive covariance (line width given by correlational value), while light purple bidirectional lines represent negative covariance. Numbers centered on directed lines indicate standardized beta coefficients and bidirectional lines indicate covariance estimate values.

The standardized beta coefficients can be seen in Figure 4 in the graph model, sharing many of the same associations as the first SEM but with the addition of the SAC and depression scales (note that the addition of new variables resulted in different associations than the first SEM in some cases with different corresponding coefficients and confidence intervals). As such, for direct effects, alexithymia was significantly positively associated with psychological inflexibility (reporting standardized beta coefficients) (β = 0.36, CI = [0.21, 0.52], *p* < 0.001); alexithymia was also significantly positively associated with autism (β = 0.27, CI = [0.10, 0.51], *p* < 0.001); alexithymia was not significantly associated with ISen self-regulation (β = −0.08, CI = [−0.26, 0.11], *p* = 0.29). OCD was significantly positively associated with psychological inflexibility (β = 0.39, CI = [0.24, 0.53], *p* < 0.001). Psychological inflexibility was no longer significantly negatively associated with ISen self-regulation (β = −0.08, CI = [−0.27, 0.08], *p* = 0.32) (this has been significantly associated in the first SEM, indicating indirect mediation effect of SAC). ISen self-regulation was significantly negatively associated with autism (β = −0.26, CI = [−0.39, −0.11], *p* < 0.001). Novel to this SEM was the introduction of an association between psychological inflexibility and SAC which was significant (β = −0.51, CI = [−0.61, −0.39], *p* < 0.001). Also, there was an association between psychological inflexibility and depression which was also significant (β = 0.60, CI = [0.51, 0.70], *p* < 0.001); a significant association between SAC and depression (β = −0.14, CI = [−0.26, −0.01], *p* < 0.05); and a significant association between SAC and ISen self-regulation (β = 0.36, CI = [0.21, 0.49], *p* < 0.001). For control measures (age and gender), age was significantly negatively associated with ISen self-regulation (β = −0.21, CI = [−0.31, −0.09], *p* < 0.001) and autism (β = 0.22, CI = [0.12, 0.32], *p* < 0.001). However, age was not significantly associated with neither psychological inflexibility (β = −0.10, CI = [−0.19, −0.00], *p* = 0.06) nor SAC (β = 0.04, CI = [−0.07, 0.13], *p* = 0.52), nor depression (β = −0.03, CI = [−0.13, 0.07], *p* = 0.51). Gender was not significantly associated with neither ISen self-regulation (β = 0.06, CI = [−0.04, 0.17], *p* = 0.32), nor autism (β = 0.11, CI = [0.00, 0.21], *p* = 0.05), nor psychological inflexibility (β = −0.01, CI = [−0.11, 0.09], *p* = 0.88), SAC (β = −0.10, CI = [−0.23, 0.01], *p* = 0.08, nor depression (β = 0.05, CI = [−0.06, 0.12], *p* = 0.36).

In terms of mediating indirect effects, age was not indirectly associated with ISen self-regulation (β = 0.04, CI = [−0.01, 0.09], *p* = 0.09) (this had been a significant indirect effect in the first SEM). However, age was significantly indirectly associated with SAC *via* psychological inflexibility (β = 0.05, CI = [0.00, 0.10], *p* < 0.05) and depression *via* psychological inflexibility and SAC (β = −0.07, CI = [−0.14, −0.01], *p* < 0.05). There were significant indirect effects between alexithymia and SAC *via* psychological inflexibility (β = −0.19, CI = [−0.28, −0.10], *p* < 0.01), alexithymia and ISen self-regulation *via* psychological inflexibility (β = −0.10, CI = [−0.21, −0.03], *p* < 0.01), and alexithymia and depression *via* psychological inflexibility and SAC (β = 0.14, CI = [0.14, 0.36], *p* < 0.01). There were also indirect effects between OCD and SAC *via* psychological inflexibility (β = −0.19, CI = [−0.29, −0.12], *p* < 0.01), OCD and ISen self-regulation *via* psychological inflexibility (β = −0.10, CI = [−0.19, −0.04], *p* < 0.01), and OCD and depression *via* psychological inflexibility and SAC (β = 0.26, CI = [0.15, 0.37], *p* < 0.01). There were also indirect effects between psychological inflexibility and ISen self-regulation *via* SAC (β = −0.19, CI = [−0.28, −0.10], *p* < 0.01), psychological inflexibility and depression *via* SAC (β = 0.07, CI = [0.01, 0.14], *p* < 0.05), and psychological inflexibility and autism *via* ISen self-regulation (β = 0.07, CI = [0.02, 0.16], *p* < 0.01. Additionally, there were indirect effects between SAC and autism *via* ISen self-regulation (β = −0.09, CI = [−0.17, −0.14], *p* < 0.01).

### Visualizing SEM in graph theory networks

A graph of nodes and edges utilizing graph theory can represent an SEM and can be modeled through available R packages, such as SEMgraph (Palluzzi and Grassi, 2021). Within a graph theory approach, an exogenous parent variable is a source node with incoming connectivity equal to 0. They are also two types of endogenous variables called “connectors” with non-zero outgoing connectivity, and another called “sinks” which have no outgoing connections. There are three types of path diagrams in graph theory: (1) directed acrylic graphs (DAG) which use beta coefficients β_*jk*_, to give a magnitude to directed edges (*k*→*j*) within the graph., but all covariances are assumed null (ψ_*jk*_ = 0); (2) covariance models in which only covariances have non-zero values and coefficients can only equal to zero (β_*jk*_ = 0); (3) bow-free acrylic graphs (BAPs), which have acrylic directed edges (*k*→*j*), and bidirectional covariance relations (*k*↔*j*) only when the *k*-th and *j*-th variable do not share a directed edge, therefore, if β_*jk*_≠0 then ψ_*jk*_ = 0. Through this graph approach, latent variables are marginalized out and instead are represented as correlations among unobserved latent confounders (Pearl, 1998).

Structural equation modeling is based on a system of structural (typically linear) regression equations that defines a path diagram that can be represented as a graph *G* = (*V, E*), whereby *V* is a set of nodes (the variables) and *E* is a set of edges that can be both directional (*k*→*j*) if *k ϵ pa*_(*j*)_ or bidirectional edges (*k*↔*j*) if *k ϵ sib*_(*j*)_, where the parent set *pa*_(*j*)_ and the sibling set *sib*_(*j*)_ determine the linear equations in the following way:


(2) $$Y_{j}\;=\;\sum_{k\;\epsilon \;pa_{\left(j\right)}}\beta_{jk}Y\;+\;U_{j}\;j\epsilon \;V$$

(3) $$Cov\left(U_{j};U_{k}\right)\;=\{\begin{array}{cc} \psi_{jk} & if\;j=k\;or\;k\;\epsilon \;sib_{\left(j\right)}\\ 0 & otherwise \end{array}$$

Where *Y*_*j*_ and *U*_*j*_ are, respectively, an unobserved variable and unobserved error term. β_*jk*_ represents a regression coefficient, and ψ_*jk*_ denotes a covariance (*Cov*) which indicates the errors are dependent when there is a latent unobserved confounder between *k* and *j*.

This is a simple graph, which has at most one edge between a pair of nodes, which are identifiable whereby parameters β and ψ can be estimated from a population covariance matrix of the observed variables (Pearl, 1998; Brito and Pearl, 2002). For the graph implementation of the second SEM with DVs of autism and depression, refer to Figure 4^7^.

### Cluster networks of SEM in graph theory to expand functional contextual properties

It is also possible to define topological communities through network clustering, and this can be done through existing R packages, such as igraph (Csardi and Nepusz, 2006). For example, there is the walktrap community detection algorithm based on random walks (Pons and Latapy, 2005), which generates as many clusters as is needed to cover the whole network. There is also the edge betweenness clustering (Newman and Girvan, 2004) that produces one large network and several other subnetworks. In addition, there is also tree agglomerative hierarchical clustering (Yu et al., 2015) for complex networks that include hierarchical properties. These algorithms are useful particularly when scaling up the network, to include additional dimensions and levels defined in the EEMM. For example, tree agglomerative hierarchical may be useful when dealing with complex multi-level networks, such as incorporating individual factors plus societal factors.

Several measures in this study were ACT consistent, such as psychological inflexibility and SAC. However, at more basic behavioral functional analytic level, relational frame theory (RFT) underpins these processes of change (Hayes et al., 2001; Blackledge, 2003; Barnes-Holmes et al., 2015). This is a post-Skinnerian behavioral model for higher cognition, related to symbolic reasoning, and based on functional contextualism. It assumes that language can be thought of as patterns of generalized contextually controlled derived relational responding. It focuses on the role of context *via* contextual cues in facilitating the emergence of patterns of relating in which functions of stimuli become connected to these patterns (Hayes et al., 2001). It assumes that relational responding can either be arbitrary or non-arbitrary. Non-arbitrary responding relates to basing responding on physical and actual features of the environment, whereas arbitrary applicable responding relates to responding being controlled by historical contextual cues. There are several patterns of arbitrary applicable responding, which include the following: coordination (e.g., stimuli x is equivalent to stimulus y), causation (IF, THEN), comparison (A is bigger than B), opposition (e.g., up is the opposite of down), distinction (e.g., C is not the same as D), hierarchy (e.g., a Labrador is a type of dog), and perspective-taking (deictic) involving the interpersonal (I vs. YOU), spatial (HERE vs. THERE) and temporal relations (NOW vs. THEN).

In total, three essential properties are important for relational frames to develop and to organize a network (Torneke, 2010): (1) mutual entailment (ME), relating one stimulus entails relating to a second stimulus; (2) combinatorial entailment (CE), relating a first stimulus to a second, and the second to a third, facilitates entailment between the first stimulus and the third; (3) the last one is the transfer (or transformation) of stimulus function (ToF) whereby the functions (e.g., fear) of any stimulus that participates within a relational frame may be transferred or transformed in line with the relations that the stimulus shares with other stimuli also participating in that frame. The individual frames can network, forming complex networks of relational frames, and can even be relationally framed with other relationally framed networks (relating relational networks) (Barnes-Holmes et al., 2017) becoming infinitely complex. Idiographic approaches of PBT such as when collecting EMA data, longitudinally, are ideal for capturing such complexity of these relational networks and how they transform (ToF) over time.

**Figure 5:**
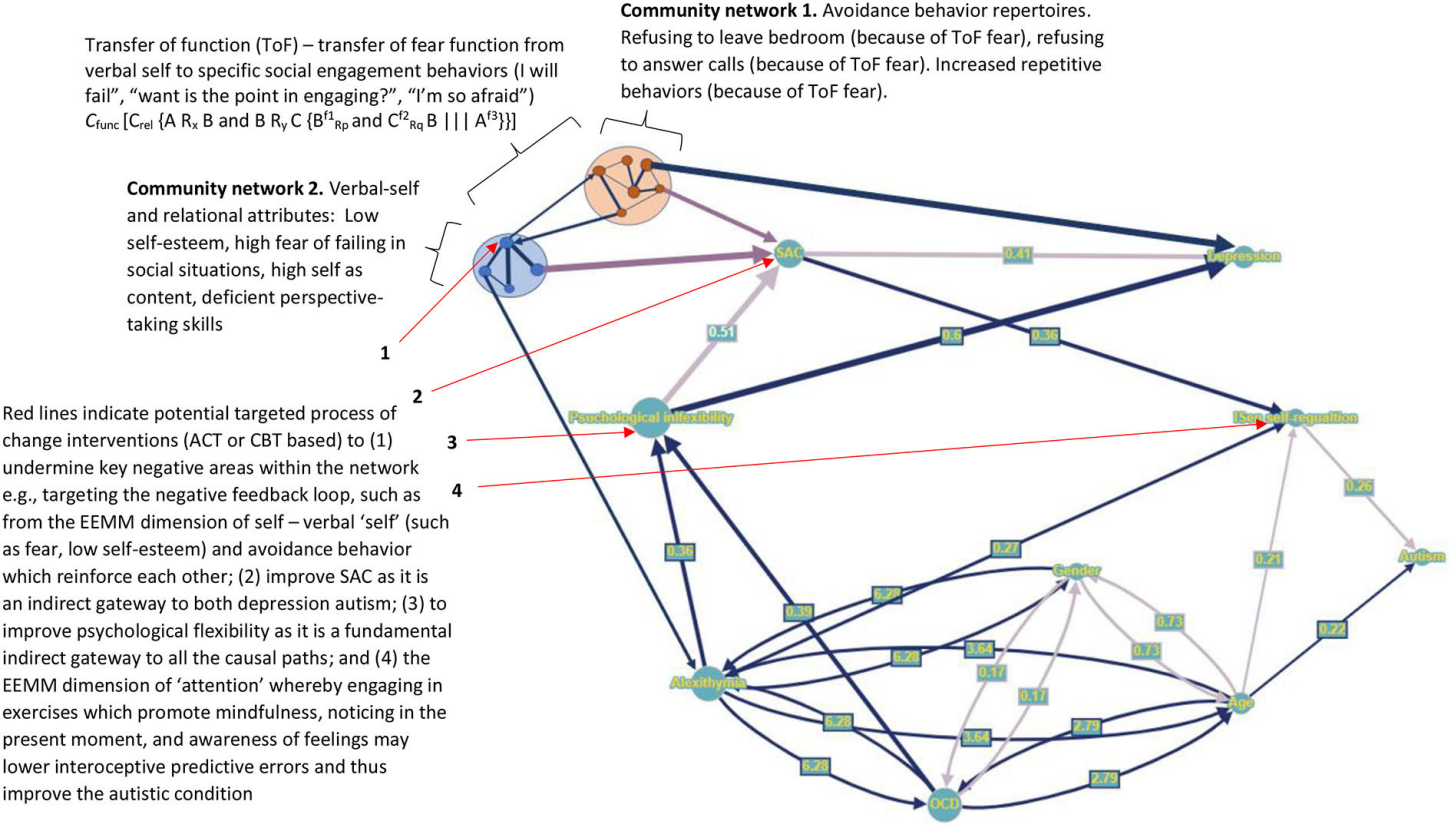
A conceptual extension of the graph nodes and edges representation in Figure 4, when extending ideographically and implemented through a more dynamic RFT PBT approach which include additional community cluster networks.

This functional analytic level of RFT applied at an ideographic level in line with EEMM and PBT could further expand the graphs (depicted in Figure 5) utilizing community cluster networks. This could allow for basic level relational frame process of change properties, such as ToF to be mapped onto a PBT graph. Some works (Smith and Hayes, 2022) have already explored this level of analysis with graphs, such as through implementing a graph model specific to RFT properties, such as ME bidirectionality. Such an approach could also allow for complex community networks (relations of relational frames) to be explored along with the traditional middle level ACT constructs of psychological flexibility and SAC. This may allow for an implementation of RFT community graphs as discussed in recent work (Edwards et al., 2022) and conceptually illustrated in Figure 5. Each node represented as a set, and therefore giving graphs the ability to represent and update in real time ME or ToF from one community set to the next, could be possible and beneficial. For example, ToF can be represented as a combination of two sets as follows:


(4) $$\begin{array}{l} C_{func}[C_{rel}\{AR_{x}B\;and\;BR_{y}C\{B_{Rp}^{f1}\;and\;C_{Rq}^{f2}B|||A^{f3}\}\}] \end{array}$$

Here → is used to show the direction of the ToF from one stimulus to another.

Where *C*_func_ is a context that controls the ToF, C_rel_ is a contextual relation, and ^f^ is a function, in this case “fear.” Here, “A” is a neutral socializing event and is related to (R_x_) you (“B”) as you try to decide will attend the socializing event, and you (“B”) are also related to (R_y_) your own verbal-self (“C,” a community network in itself which relationally frames many thoughts and feelings about yourself, and its dominated by fear, thoughts about failure, and low self-esteem). THEN you (B) become fearful (${}_{\text{Rp}}^{\text{f}1}$) of the social event (A) as the function of A (A^f3^) transforms from neutral to fear (${}_{\text{Rq}}^{\text{f}2}$) (C → A ToF) as your feelings of failure about yourself, and fear, become mutually entailed (|||) with the social event, (as you think about failing in socializing at this event, and how scared you will be) which now shares the fear function A^f3^ of your verbal self (C^f2^). This leads the individual to decide to avoid the social situation (A), to prevent further pain of fear and failure thus causing the individual to isolate further.

These community networks of verbal self and the resultant avoidance behaviors (and their associated relational frames) not only act to transfer a fear function, but they act to reinforce one another. Low self-esteem and fear of the verbal self lead to a greater probability of avoidance (isolation from others due to ToF), and the resultant avoidance then further reinforces low self-esteem and fear of the verbal self (as you may tell yourself that you have again failed), which then increases the chances of further avoiding social events (and communicating more generally) in the future. This is called a negative feedback loop (or a self-reinforcing loop of maladaptation) in PBT (Hayes and Hofmann, 2020; Hofmann et al., 2021). It is suggested that these should be identified and targeted with the correct interventions (ACT or CBT) (as seen in Figure 5 for an example) to undermine this negative feedback loop.

Such modeling at the level of the relational frame could ultimately give the PBT therapist very strong influence over the entire network, thus making them more able to influence processes of change at a basic functional contextual level, in a dynamic way which is consistent with EEMM. This approach is more relevant at the ideographic level as individuals each form their own unique relational networks through their unique learning histories. It could also be extended even further with an RFT implemented reinforcement machine learning interpretation through discrete time series Markov chains (Edwards, 2021) to analyze reinforced behavior (and any other EMA data) through time in a complex distributed system (i.e., the real-world environment, and outside of the laboratory) (Dabrowski and Hunt, 2011).

## General discussion

This study aimed to identify and confirm two conceptual models through two corresponding SEMs, which maps the causal pathways between alexithymia and OCD (as exogenous IVs) with autism and autistic-related depression (as endogenous DVs) *via* the variables bodily interoception, psychological inflexibility, and SAC (with age and gender as controls).

To do this, the first part of this study explored through ANCOVA whether age had an effect on autonomic nervous functioning in healthy controls. The measure for this was HRV (HRV is closely correlated with the ISen interoceptive measures used in the questionnaires), and BMI and gender were added as covariates. There was a significant decrease in HRV given age, and this finding highlighted the importance of controlling the SEM for age, as it would likely be a significant control in the SEMs and having a direct effect on autonomic functioning (ISen interoception) within the network regardless of the autistic condition. Within the SEM, age was found to significantly affect both ISen self-regulation interoception and autism directly as expected, though the indirect effect between age and autism *via* ISen self-regulation interoception was non-significant.

The second part of the study's goal was to develop a conceptual SEM. However, the literature gave limited clues as to what the structure of the SEM should consist of (other than demonstrating there was much covariation between OCD, alexithymia, and autism). Recent work (Edwards and Lowe, 2021) had given some clues in how alexithymia relates to psychological inflexibility and SAC, but not in the cases of covarying OCD and autism. So, to explore this further, three linear (backward) regressions were explored, to first (in the first regression model) determine whether the measures of mental health, OCD, interoception (ISen), alexithymia, psychological inflexibility, and self-as-context predicted ASD as an outcome. The second regression model was used to determine whether the measures of mental health, OCD, interoception (ISen), autism, psychological inflexibility, and self-as-context predicted alexithymia. The third regression model was used to determine whether the measures of mental health, alexithymia, interoception, autism, psychological inflexibility, and self-as-context predicted OCD. The outcomes in the final regression step models informed the third part of the study in terms of which variables were most likely to directly predict each of the three variables (OCD, alexithymia, and autism).

One problem with the regression models is that they only show direct associations and give no clues how the variables may indirectly associate with one another. In the third part of the study, five independent simple mediation analyses were conducted to give some clues about how these variables indirectly relate to one another. Here, some of the outcomes in the final regression models (of part two) informed the mediation analyses to be made. The first mediation analysis showed that ISen attention regulation partially mediated the relation between OCD and autism. The second mediation analysis showed that SAC partially mediated the relation between autism and alexithymia. The third showed that ISen trusting partially mediated the relation between alexithymia and depression. The fourth showed that psychological inflexibility partially mediated the relation between ISen attention regulation and SAC. The fifth showed that psychological inflexibility partially mediated the relation between SAC and ISen trusting. What is interesting about these mediation analyses is that at a hierarchical level (as depicted in Figure 1), psychological inflexibility seems to at least partially mediate all of the different relations at the highest hierarchical level. This provided some insights into a conceptual model, indicating that psychological inflexibility should be a key indirect pathway between the IVs (OCD and alexithymia) and DVs (autism and autistic-related depression).

In the fourth part of the study, the conceptual models for causal pathways were developed concretely, whereby IVs were identified as OCD and covarying alexithymia, while the DVs were autism (in model one) and autistic-related depression (in model two, with autism as a DV). These were tested through two SEMs, but before this could happen, the first steps were to utilize EFA and CFA to assign (explore then confirm) item variables to latent constructs where possible. However, when utilizing these factor analysis approaches items within the same questionnaire constructs did not always load well together. Items variables were removed from the latent variables when the standardized residual covariances were significantly different from the data to improve the overall SEM fit. This meant that items such as the many subsections of the ISen questionnaire did not fit well together in the context of the data and conceptual model and were removed when they did not. Once these were removed, the final first SEM that included autism only as a DV showed that alexithymia was directly significantly positively associated with autism and psychological inflexibility, but it (alexithymia) was not directly significantly associated with ISen self-regulation. Alexithymia was instead indirectly associated with ISen self-regulation *via* psychological inflexibility. OCD was not directly or indirectly associated with autism, but it was indirectly associated with ISen self-regulation mediated by psychological inflexibility. Psychological inflexibility was not directly associated with autism, but it was indirectly associated with it *via* ISen self-regulation. Gender was not a significant control, but age was a strong controlling variable (as expected given the data from the ANCOVAs). Age was directly associated (a direct control) to autism and was significantly negatively associated with ISen self-regulation directly and indirectly *via* psychological flexibility. Age was marginally outside of significance in its association (control) with psychological inflexibility directly.

In the second SEM, these findings were extended further, by including a second DV in the form of depression. Psychological inflexibility was directly associated with depression and indirectly associated with it *via* SAC, while SAC was directly associated with depression. Neither age nor gender has any direct associations with SAC or depression. Age was significantly indirectly associated with SAC *via* psychological inflexibility and depression *via* psychological inflexibility and SAC. There were also indirect effects between alexithymia and SAC *via* psychological inflexibility, alexithymia and ISen self-regulation *via* psychological inflexibility, and alexithymia and depression *via* psychological inflexibility and SAC. There was an indirect effect between OCD and SAC *via* psychological inflexibility, OCD and ISen self-regulation *via* psychological inflexibility, and OCD and depression *via* psychological inflexibility. There were indirect effects between psychological inflexibility and ISen self-regulation *via* SAC, psychological inflexibility, and depression *via* SAC, and between SAC and autism *via* ISen self-regulation.

Perhaps, the most important findings in this study are that the SEMs show the clear indirect causal association relation between alexithymia and OCD with autism. Though alexithymia and OCD shared a strong correlation with autism, the model confirmed that the best fit of the data were through an indirect causal association with autism, indirectly through psychological inflexibility and SAC and then indirectly through ISen self-regulation interoception. This indicates that there exists a complex relation between psychological flexibility and vagal nerve related interoception, and this is supported by an increasing number of evidence (Pinna and Edwards, 2020; Edwards and Lowe, 2021; O'Brien et al., 2021). This could even include connections with embodied knowledge of psychological flexibility (Falletta-Cowden et al., 2022) as the ISen interoceptive measure used relates to confidence in embodied interoceptive signals. The findings also suggest that these complex associations are causally related to autism, and this is not captured in the current diagnostic DSM protocol. The SEMs show causally how alexithymia and OCD are comorbidly showing up with autism.

The findings also show that autistic-related depression is caused indirectly through psychological inflexibility and SAC, and there was no direct causal path between depression and autism (despite there being a strong correlation between depression and autism). Much of the causal pathway traffic in the associations between alexithymia and autism and its associated depression was *via* psychological inflexibility. The causal diagram shows psychological inflexibility to be a central gateway to the link between alexithymia and OCD with autism (which was predicted from the results of the mediational analysis, in the third part of the study given its hierarchical position), whereby ISen self-regulation bodily interoception is a secondary gateway for the link between alexithymia and OCD with autism severity specifically. SAC association with psychological inflexibility and autistic-related depression also seems a key secondary causal path gateway for the depression specifically. As SAC relates to perspective-taking skills, it seems likely that the onset of depression is linked to the lack of ability of ASD individuals to connect and communicate with others (for which perspective-taking skills is crucial). Building such perspective-taking skills may be a key and useful intervention in reducing depression in autism, given this nomothetic model.

This SEM path analysis helps to delineate the associations between alexithymia, OCD, and ASD more concretely. This highlights why these conditions cannot be taken as singular discrete categorical syndromes with an independent set of symptoms and underlying etiology which is treatable through a static protocol for treatment as specified by the DSM (American Psychiatric Association, 2013a). Instead, this study results support the claim that there is great comorbidity between these conditions (Kupfer et al., 2008) whether direct or indirectly. They also support the claim that any specified singular treatment protocol (such as what is specified by the DSM) would lack treatment specificity (Kupfer et al., 2008; Insel et al., 2010). Given such comorbidity, as seen in the casual diagram, any individual changes, or differences at any of the intersecting indirect pathways, could lead to quite different outcomes and have cascading effects. Therefore, a more dynamic and ideographic (individual level) process-based approach is likely to be more effective to access and initiate processes of change within then complexity, rather than a strict and rigid DSM protocol from treatment in treating individuals with autism.

This work supports the conclusions made by PBT and EEMM research advocates that suggest dynamic, longitudinal, and multi-level (e.g., biological and psychological) approaches are required to identify important change processes rather than singular protocols for syndromes (Hayes and Hofmann, 2017, 2018; Hofmann and Hayes, 2019; Hofmann et al., 2021). The SEM presented here (though nomothetic data) fits well with the EEMM as it emphasizes that autism is a multi-level condition. For example, it identifies that interoception (ISen self-regulation) fits well with both the individual level dimension of “attention” as it relates to noticing bodily feelings and the level of “physiology” as the vagal nerve carries bodily information to the brain and is processed in the insular cortex. SAC fits with the “self” dimension and psychological flexibility with the “cognitive” dimension at the individual level.

In relation to the theories of interoception more generally, interoception defined as a predicting coding error (Seth, 2013; Barrett and Simmons, 2015; Barrett et al., 2016; Seth and Friston, 2016; Stephan et al., 2016; Owens et al., 2018) overcomes some of the issues with directional predictions typically associated with interoception. Higher ISen self-regulation interoception represents high confidence in the interoceptive signals, and therefore, this is consistent with the predictive coding theory, as low confidence should mean high prediction errors. This interpretation of interoception suggests that autism is a condition whereby there are high bodily prediction errors. To lower these predictive errors using the PBT EEMM approach, the “attentional” dimension may be an important domain (as illustrated in intervention point 4 of Figure 5), whereby engaging in exercises which promote mindfulness, noticing in the present moment, and awareness of feelings may lower interoceptive predictive errors and thus improve the autistic condition. Again, this would be most accurately done through ideographic assessments, such as EMA longitudinally overt time giving precise ever-changing interoceptive predictive coding data within the complex network.

In relation to ASD, the predictive coding model explains why autistic individuals tend to focus on local information (ignoring contextual prior background knowledge) as it suggests that the predictive priors of contextual background information are weaker in ASD individuals when compared to healthy individuals, as observed in previous studies (Happé and Frith, 2006; Mottron et al., 2006). As such when developing a longitudinal EMA PBT study, we would expect to see contextual relational frame community clusters (as conceptually illustrated in Figure 5) more heavily weighed to recent events. This would mean, for example, something recently learned, i.e., whereby a function in the network transfers from one stimuli of a cluster to the next (through ToF), then the impact would be greater for more local recent events than something learned further back in time. This would perhaps mean targeting the verbal self with a cognitive defusion intervention to undermine the negative feedback loop of avoidance (as illustrated in intervention point 1 of Figure 5) resultant from destructive verbal self-language (e.g., “I will fail”), thus preventing such negative transformations of functions. It could also mean targeting SAC with perspective-taking building skills to help the individual communicate and see others perspective (as illustrated in intervention point 2 of Figure 5). Crucially, it should mean targeting the key gateway of the network, psychological inflexibility such as through values orientation, mindfulness, openness to pain exercises, and cognitive defusion (as illustrated in intervention point 3 of Figure 5).

The advantage of the longitudinal approach advocated by the PBT developers (Hayes and Hofmann, 2017, 2018; Hofmann and Hayes, 2019; Hofmann et al., 2021) is that this longitudinal and ideographic approach would allow therapists and researchers enough detail (data) to model these types of transformations of functions in real time and ideographically for each individual. Existing graph approach such as group iterative multiple model estimation (GIMME) approach (Gates and Molenaar, 2012; Beltz and Gates, 2017; Weigard et al., 2021), which combines the elements of graph theory with SEM, is useful for modeling ideographic longitudinal EMA type data and is advocated by PBT researchers (Hayes et al., 2019). However, these may be further developed in line with an ideographic version of what is proposed in Figure 5, which would help to account for dynamic and ongoing relational frame processes, such as ToF. This may be further helpful for clinicians and researchers alike and would allow for interventions to target and undermine these negative functions directly and affect the overall network at the level of the relational frame.

All studies include limitations. Questionnaire data in this study required participants to answer all questions, so this may have impacted attrition rates as some may have dropped out of the study due to frustration. Another limitation was that the data used here were solely cross-sectional nomothetic (population-based). However, PBT approaches are optimized when exploring longitudinal ideographic data, such as through EMA, therefore, it would be useful to explore this SEM in a longitudinal and ideographic way to see whether the model is still supported by the data. Finally, ideally, a physiological measure such as HRV would have supplemented the interoceptive measure greatly, though these are related.

In conclusion, this study demonstrated that autism is a complex and dynamic condition, which is not easily definable and is in part caused by alexithymia and OCD indirectly through psychological inflexibility, SAC, and interoception (ISen self-regulation). Though some of these associations are quite predictable such as covarying OCD with alexithymia, the novelty of this study was to capture the complex processes (psychological inflexibility, SAC, and interoception) that indirectly related to OCD, alexithymia, and autism. Further studies exploring the ideographic level and longitudinally may be able to generate more complex graphs that can model specific relational frame processes, such as ToF and dynamically in real time, which may greatly benefit both researchers interested in PBT and therapists utilizing this approach to support autistic individuals.

## Data availability statement

The raw data supporting the conclusions of this article will be made available by the authors, without undue reservation.

## Ethics statement

The studies involving human participants were reviewed and approved by Swansea University Psychology Ethics. The patients/participants provided their written informed consent to participate in this study.

## Author contributions

DE completed all aspects of this manuscript.

## Conflict of interest

The author declares that the research was conducted in the absence of any commercial or financial relationships that could be construed as a potential conflict of interest.

## Publisher's note

All claims expressed in this article are solely those of the authors and do not necessarily represent those of their affiliated organizations, or those of the publisher, the editors and the reviewers. Any product that may be evaluated in this article, or claim that may be made by its manufacturer, is not guaranteed or endorsed by the publisher.

## Footnotes

^1^Full details can be found at: https://physionet.org/content/autonomic-aging-cardiovascular/1.0.0/.

^2^Full details can be found at: https://physionet.org/content/autonomic-aging-cardiovascular/1.0.0/.

^3^See here for Qualtrics: www.qualtrics.com.

^4^See here for full procedural details: https://physionet.org/content/autonomic-aging-cardiovascular/1.0.0/.

^5^See here for full details: https://physionet.org/content/autonomic-aging-cardiovascular/1.0.0/.

^6^See here for G^*^Power software: https://stats.idre.ucla.edu/other/gpower/.

^7^Graph developed with software found at: https://graphonline.ru/.
